# Supplementary material for: Effects of treated wastewater on the ecotoxicity of small streams – Unravelling the contribution of chemicals causing effects
Source: PLoS One. 2019 Dec 27;14(12):e0226278. doi: 10.1371/journal.pone.0226278 (PMC6934383; doi:10.1371/journal.pone.0226278)
Supplement: S1 Appendix — (PDF) [file pone.0226278.s001.pdf]

Effects of treated wastewater on the ecotoxicity of small streams – unravelling the contribution of chemicals causing effects

Supporting information

Cornelia Kienle[1*], Etiënne L. M. Vermeirssen[1], Andrea Schifferli[1], Heinz Singer[2], Christian Stamm[2], Inge Werner[1]

[1] Swiss Centre for Applied Ecotoxicology, Dübendorf, Zürich, Switzerland

[2] Department of Environmental Chemistry, Swiss Federal Institute of Aquatic Science and Technology (Eawag), Dübendorf, Zürich, Switzerland

*Corresponding author

E-Mail: cornelia.kienle@oekotoxzentrum.ch

Table of Contents

S1 Information on the sampling locations 6

S2 Information on dilution coefficients 9

S3 Sample preparation procedure for chemical analysis of estrogenic compounds and for bioassays 10

S4 Material and methods 11

- **S4.1 *Gammarus fossarum* feeding activity** 11
- **S4.2 *Ceriodaphnia dubia* reproduction assay** 12
- **S4.3 ERα-CALUX®** 13

S5 Equations for bioassay data analysis 14

S6 Relative effect potencies for the *in vitro* bioassays 15

S7 Applied annual average environmental quality standards (AA-EQS) for risk assessment based on individual chemicals 17

S8 Results 19

- **S8.1 General water chemistry** 19
- **S8.2 Micropollutant screening** 19
- **S8.3 Results of *in vitro* bioassays** 23
- **S8.4 Results of ERα-CALUX®** 25
- **S8.5 *In vitro* bioassays and dissolved organic carbon** 29
- **S8.6 Adding compounds below LOQ as LOQ/2** 30
- **S8.7 *In vivo* bioassays** 33
- **S8.8 Correlation of effects measured in effluents and river water** 35
- **S8.9 Mixture risk assessment based on bioassay results** 38
- **S8.10 Mixture risk assessment based on chemical analysis results** 40

List of Tables

Table A: Sampling sites with information on wastewater treatment plants and land use percentage (from Munz, Burdon [1]). 6

Table B: Sampling overview (adapted from Munz, Burdon [1]) 7

Table C: Dilution coefficients, calculated based on all measured general water quality parameters from the respective sampling day. 9

Table D: Solid phase extraction for chemical analysis of estrogenic compounds and for bioassays. 10

Table E: Specifications of chemical analysis of estrogenic compounds using Liquid Chromatography Mass Spectrometry (LC-MS/MS). 11

Table F: 17β-estradiol equivalence factors (EEF) for estrone (E1), 17β-estradiol (E2), 17α-ethinylestradiol, bisphenol A und nonylphenol. Literature values. 15

Table G: Diuron equivalence factors (DEF) for photosystem II inhibiting herbicides in the combined algae assay. 15

Table H: Parathion equivalence factors (PtEF) for acetylcholinesterase inhibiting insecticides in the acetylcholinesterase inhibition assay. 16

Table I: Annual average environmental quality standards (AA-EQS, ng/L) for the individual estrogenic compounds. 17

Table J: Annual average environmental quality standards (AA-EQS, ng/L) for the individual photosystem II-inhibitors (adapted from [15]). 17

Table K: Annual average environmental quality standards (AA-EQS, ng/L) for the acetylcholinesterase inhibitors (adapted from [15]). 18

Table L: Summary of results from estrogen measurements (ng/L). 19

Table M: Summary of results from measurements of photosystem II inhibitors (ng/L) 20

Table N: Summary of results from measurements of acetylcholinesterase inhibitors (ng/L). 22

Table O: Summary of results from the Yeast Estrogen Screen (YES) and the ERα-CALUX®. 23

Table P: Summary of results from the combined algae assay. 23

Table Q: Summary of results from the acetylcholinesterase inhibition assay. 24

List of Figures

Fig A: Overview on sampling sites from 2013 and 2014. n = 24 8

Fig B: Overview on one study site. 8

Fig C: Correlation of 17β-estradiol equivalent concentrations (EEQ_{bio}, ng/L) in the Yeast Estrogen Screen (YES) and the ERα-CALUX® at 12 sites investigated in 2014 in wastewater treatment plant (WWTP) effluent as well as in the river up- and downstream of the WWTP discharge. 25

Fig D: Estrogenic activity in the ERα-CALUX®: 17β-estradiol equivalent concentrations (EEQ_{bio}, ng/L) at 12 sites investigated in 2014 in wastewater treatment plant (WWTP) effluent as well as in the river up- and downstream of the WWTP discharge. 26

Fig E: Correlation of 17β-estradiol equivalent concentrations measured in the ERα-CALUX® (EEQ_{bio}, ng/L) to the values calculated by chemical analysis (EEQ_{chem}, ng/L) based on relative potencies of the measured estrogens in the bioassay. 27

Fig F: Contribution of individual estrogenic compounds measured in chemical analysis (EEQ_{chem}, based on relative potencies of the measured estrogenic compounds in the bioassay) to the 17β-estradiol equivalent concentrations (EEQ_{bio}, ng/L) measured in the ERα-CALUX®. 28

Fig G: Correlation of (A) 17β-estradiol equivalent concentrations (EEQ_{bio}, ng/L) measured in the Yeast Estrogen Screeen, (B) diuron equivalent concentrations (DEQ_{bio}, ng/L) measured in the combined algae assay, and (C) parathion equivalent concentrations ($PtEQ_{bio}$, ng/L) measured in the acetylcholinesterase inhibition assay to dissolved organic carbon (DOC) values (mg/L) measured in the water samples. 29

Fig H: Correlation of bioanalytical equivalent concentrations measured in (A) the Yeast Estrogen Screen (YES, 17β-estradiol equivalent concentrations, EEQ_{bio}, ng/L), (B) the ERα-CALUX® (EEQ_{bio}, ng/L), (C) the combined algae assay (diuron equivalent concentrations, DEQ_{bio}, ng/L), and (D) the acetylcholinesterase (AChE) inhibition assay (parathion equivalent concentrations, $PtEQ_{bio}$, ng/L) to the values calculated by chemical analysis (EEQ_{chem}, DEQ_{chem}, $PtEQ_{chem}$, resp.) based on relative potencies of the measured estrogens, PSII inhibitors or AChE inhibitors in the bioassays. Values below LOQ were included as LOQ/2. 30

Fig I: Contribution of individual estrogens measured in chemical analysis (EEQ_{chem}, calculated based on relative potencies of the measured estrogens in the bioassay) to the 17β-estradiol equivalent concentrations (EEQ_{bio}, ng/L) measured in the Yeast Estrogen Screen (YES). Values below LOQ were included as LOQ/2. 31

Fig J: Contribution of individual PSII inhibitors measured in chemical analysis (DEQ_{chem}, based on relative potencies of the measured PSII inhibitors in the bioassay) to the diuron equivalent concentrations (DEQ_{bio}, ng/L) measured in the combined algae assay. Values below LOQ were included as LOQ/2. 32

Fig K: Number of offspring of *Ceriodaphnia dubia* after 7 days exposure to river water from 4 sites investigated in 2013. 33

Fig L: Feeding rate of *Gammarus fossarum* (mg / mg Gammarus / d) after 7 days exposure up- (US) and downstream (DS) of three sites in 2013 (Buttisholz, Hochdorf, Sévery). 34

Fig M: Correlation of 17β-estradiol equivalent concentrations (EEQ_{bio}, ng/L) measured downstream in the river in (A) the Yeast Estrogen Screen (YES) and (B) the ERα-CALUX® to the values calculated by measurements in the wastewater multiplied by the respective dilution factor in the river minus the EEQ values measured in the river upstream of the WWTP. C and D display the fraction of wastewater measured using general water chemistry data and calculated based on results of the YES (C) and the ERα-CALUX® (D). 35

Fig N: Correlation of diuron equivalent concentrations (DEQ_{bio}, ng/L) for (A) photosystem II inhibition and (B) growth inhibition measured in downstream samples in the combined algae assay after 2 h and 24 h respectively to the values calculated by measurements in the wastewater multiplied by the respective dilution factor in the river minus the DEQ_{bio} values measured in the river upstream of the WWTP. C and D display the fraction of wastewater measured using general water chemistry data and calculated based on results for PSII inhibition (C) and growth (D). 36

Fig O: (A) Correlation of parathion equivalent concentrations ($PtEQ_{bio}$, ng/L) measured in samples taken downstream in the river to the values calculated by measurements in the wastewater multiplied by the respective dilution factor in the river minus the $PtEQ_{bio}$ values measured in the river upstream of the WWTP. (B) Fraction of wastewater measured using general water chemistry data and calculated based on the bioassay values. 37

Fig P: Summary risk quotients calculated from bioassay results for four substance groups (estrogenic compounds, algae PSII inhibitors, algae growth inhibitors, acetylcholinesterase inhibitors) measured in the Yeast Estrogen Screen (YES), the ERα-CALUX®, the combined algae assay, and the acetylcholinesterase (AChE) inhibition assay respectively. 38

Fig Q: Risk quotients calculated from bioassay results for four substance groups (estrogenic compounds, algae PSII inhibitors, algae growth inhibitors, acetylcholinesterase inhibitors) at 24 sampling sites in A) the river upstream and B) the river downstream of the WWTP discharge. 39

Fig R: Mixture risk quotients (RQ_{mix}) calculated from chemical analysis of photosystem II inhibitors at 24 sampling sites in A) the river upstream and B) the river downstream of the WWTP discharge over 8 sampling events (12 sites per sampling event). 40

Fig S: Mixture risk quotients (RQ_{mix}) calculated from chemical analysis of acetylcholinesterase inhibitors at 24 sampling sites in A) the river upstream and B) the river downstream of the WWTP discharge over 8 sampling events (12 sites per sampling event). 41

S1 Information on the sampling locations

Table A: Sampling sites with information on wastewater treatment plants and land use percentage (from Munz, Burdon [1]).

Site Code	Site/ WWTP name	Year	X-coordinate	Y-coordinate	Connected inhabitants[1]	Design size (PE)[1]	Treatment type[1,2]	Hydrological catchment size (km^2)[3]	Fraction arable land[3]	Fraction urban settlement[3]	Fraction meadows[3]	Fraction woods[3]	Fraction unproductive area[3]
1	Buttisholz	2013	648220	218280	2767	3500	A	6.8	42%	14%	32%	11%	0.4%
2	Colombier	2013	526325	156495	835	1066	B	11.8	81%	8%	4%	7%	0.4%
3	Dürnten	2013	705278	236123	5931	7000	I	17.4	16%	21%	44%	17%	1.8%
4	Herisau	2013	737780	250800	16155	33333	F	16.3	0.8%	20%	54%	24%	0.6%
5	Hochdorf	2013	663850	225450	11535	50000	F	28.1	47%	14%	26%	13%	0.5%
6	Hornussen	2013	646200	261550	3195	3133	H	37.0	29%	8%	20%	43%	0.5%
7	Kernenried	2013	607675	211625	28152	51750	H	65.9	43%	18%	8%	29%	1.4%
8	Messen	2013	601470	217310	5711	11666	E	37.4	50%	7%	11%	32%	0.8%
9	Niederdorf	2013	624173	251433	5355	12133	E	25.1	12%	8%	33%	46%	0.3%
10	Romont	2013	561400	172550	11063	15000	E	47.6	35%	10%	39%	16%	0.3%
11	Rothenturm	2013	693450	218620	2062	1350	E	7.4	0.1%	6%	47%	46%	1.3%
12	Sévery	2013	523600	158610	1035	1933	E	7.2	32%	6%	10%	52%	0.1%
13	Aadorf	2014	709700	262000	13193	18000	H	35.0	16%	14%	32%	36%	1.1%
14	Birmensdorf	2014	674315	246158	21437	28750	E	46.7	22%	16%	21%	38%	2.4%
15	Elgg	2014	706557	262026	3614	5633	H	13.3	27%	13%	19%	41%	0.4%
16	Ellikon	2014	704475	269615	6366	9000	H	24.1	55%	14%	11%	19%	0.7%
17	Knonau	2014	677051	230617	5457	8750	H	16.6	41%	14%	24%	20%	1.2%
18	Marthalen	2014	690284	275205	3680	6666	H	26.5	54%	13%	10%	21%	2.3%
19	Muri	2014	668300	237150	7617	11666	I	15.5	43%	15%	23%	19%	0.4%
20	Reinach	2014	655430	234850	31365	45000	H	43.6	44%	15%	20%	20%	0.5%
21	Unterehrendingen	2014	667900	262250	10299	15000	H	30.3	39%	14%	20%	27%	0.4%
22	Val-de-Ruz	2014	561680	211070	10948	-	F	63.7	17%	5%	34%	43%	0.3%
23	Villeret	2014	568550	223400	6742	8500	E	63.0	5%	7%	48%	40%	0.4%
24	Zullwil	2014	611376	249614	1182	3933	A	7.1	7%	9%	39%	45%	0.1%

[1]http://www.bafu.admin.ch/wasser/13462/13496/15866/index.html?lang=en

[2]A: Mechanical-biological treatment, B: Mechanical-biological treatment with P-elimination, E: Mechanical-biological treatment with P-elimination and nitrification, F: Mechanical-biological treatment with advanced P-elimination and nitrification, H: Mechanical-biological treatment with P-elimination, nitrification and denitrification, I: Mechanical-biological treatment with advanced P-elimination, nitrification and denitrification (VSA categories: www.vsa.ch)

[3]BFS (2014). The defined land use categories include the following sub-classes: i) urban settlement: industrial and commercial areas, building areas, transportation areas, special urban areas, recreational areas and cemeteries, ii) agriculture: orchard, vineyard and horticulture areas, arable land, iii) meadows: meadows, farm pastures, alpine agricultural areas, iv) woods: forest, brush forest, woods, v) unproductive areas: lakes, rivers, unproductive vegetation, bare land, glaciers, perpetual snow.

Table B: Sampling overview (adapted from Munz, Burdon [1])

Ecoimpact campaign	Timepoints		Sites		Organic substances analysed	Chemical Analytics	Ecotoxicological bioassays
2013	June 2013	12.06.2013	1-12: up, down	2, 10, 12	389 + 5 estrogenic compounds	Offline SPE; LC-HRMS<br>LC-MS/MS for estrogenic compounds<br>general water quality parameters	At all sites :<br>Yeast Estrogen Screen<br>Combined algae assay<br>Acetylcholinesterase inhibiton assay<br>At sites 3, 4, 6, 11: *Ceriodaphnia dubia* reproduction assay<br>At sites 1, 4, 5, 12: *Gammarus fossarum* feeding assay
		17.06.2013		7, 8, 9			
		19.06.2013		1, 5, 6, 11			
		24.06.2013		3, 4			
	February 2014	04.02.2014	1-12: up, down, effluent	2, 10, 12	57	Online SPE; LC-HRMS<br>general water quality parameters	
		05.02.2014		6, 7, 8, 9			
		18.02.2014		3, 4, 11			
		20.02.2014		1, 5			
2014	March 2014:	11.03.2014	13-24: up, down, effluent	13, 15, 16, 18, 21	57 +10 heavy metals	Online SPE; LC-HRMS<br>LC-MS/MS for estrogenic compounds<br>HR-ICP-MS for heavy metals<br>general water quality parameters	
		12.03.2014		22, 23, 24			
		19.03.2014		14, 17, 19, 20			
	May 2014:	05.05.2014	13-24: up, down, effluent	13, 15, 16, 18	up, down: 389<br>effluent: 57<br>+ 5 estrogenic compounds<br>+10 heavy metals		At all sites :<br>Yeast Estrogen Screen<br>Combined algae assay<br>Acetylcholinesterase inhibiton assay
		19.05.2014		14, 17, 19, 20			
		12.05.2014		21, 22, 23, 24			
	July 2014	30.06.2014	13-24: up, down, effluent	15, 16, 18	57 +10 heavy metals		
		01.07.2014		21, 22, 23, 24			
		05.08.2014		13, 20			
		18.08.2014		14, 17, 19			
	September 2014	15.09.2014	13-24: up, down, effluent	13, 15, 16, 18	57 +10 heavy metals		
		17.09.2014		22, 23, 24			
		18.09.2014		21			
		23.09.2014		14, 17, 19, 20			
	November 2014	05.11.2014	13-24: up, down, effluent	13, 15, 16, 18	57 +10 heavy metals		
		26.11.2014		14, 17, 19, 20			
		01.12.2014		21			
		02.12.2014		22, 23, 24			
	January 2015	19.01.2015	13-24: up, down, effluent	22, 23	57 +10 heavy metals		
		20.01.2015		14, 19, 20			
		21.01.2015		13, 15, 16, 18			
		29.01.2015		17, 21, 24			

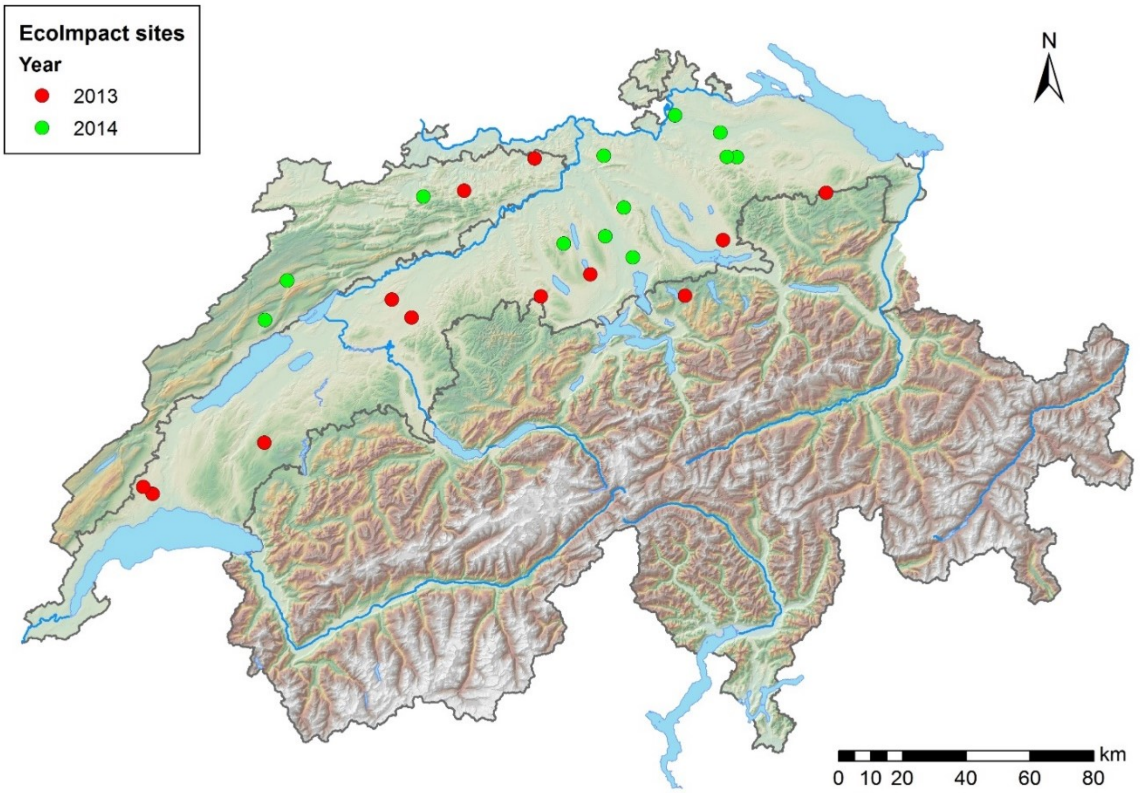

Fig A: Overview on sampling sites from 2013 and 2014. n = 24

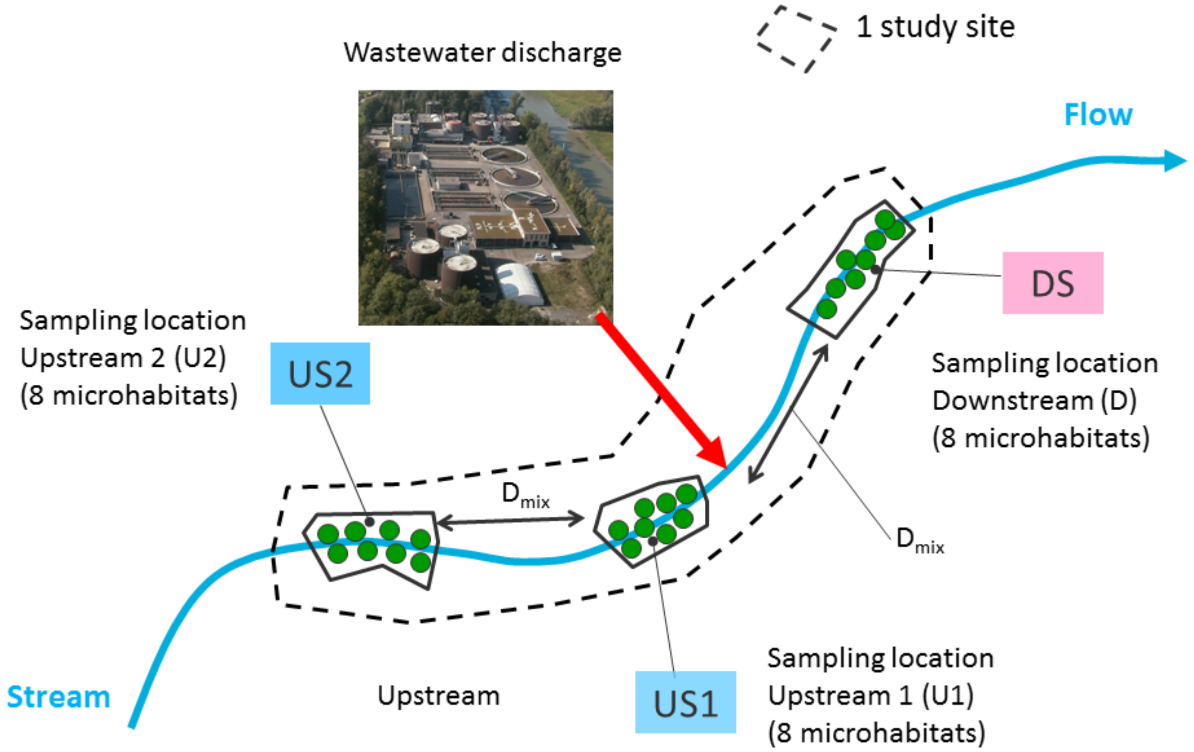

Fig B: Overview on one study site.

Upstream site 1 (US) and downstream site (DS) were sampled for the current study. Copyright/Author: Christoph Lüthi, Sandec and Eawag.

S2 Information on dilution coefficients

Table C: Dilution coefficients, calculated based on all measured general water quality parameters from the respective sampling day.

Results for general water quality parameters are presented in Burdon, Reyes [2].

Site Code	Site/ WWTP name	Year	Month	Median	Std. dev. of median
1	Buttisholz	2013	June	0.081	0.152
2	Colombier	2013	June	0.068	0.080
3	Dürnten	2013	June	0.275	0.123
4	Herisau	2013	June	0.329	0.248
5	Hochdorf	2013	June	0.127	0.089
6	Hornussen	2013	June	0.053	0.200
7	Messen	2013	June	0.053	0.059
8	Moossee-Urtenenbach	2013	June	0.294	0.165
9	Niederdorf	2013	June	0.074	0.236
10	Romont	2013	June	0.056	0.113
11	Rothenthurm	2013	June	0.021	0.035
12	Sévery	2013	June	0.029	0.012
13	Aadorf	2014	May	0.165	0.056
14	Birmensdorf	2014	May	0.225	0.071
15	Elgg	2014	May	0.083	0.014
16	Ellikon	2014	May	0.182	0.060
17	Knonau	2014	May	0.279	0.043
18	Marthalen	2014	May	0.118	0.248
19	Muri	2014	May	0.230	0.090
20	Reinach	2014	May	0.267	0.187
21	Sevaru	2014	May	0.361	0.095
22	Unterehrendingen	2014	May	0.238	0.091
23	Villeret	2014	May	0.098	0.107
24	Zullwil	2014	May	0.097	0.051

S3 Sample preparation procedure for chemical analysis of estrogenic compounds and for bioassays

Table D: Solid phase extraction for chemical analysis of estrogenic compounds and for bioassays.

	Estrogenic compounds	Bioassays
General Information		
Sample type	water samples	water samples
Sample volumes	500 mL wastewater effluent	500 mL wastewater effluent<br>1000 mL river water
Blank	500 mL ultrapure water	1000 mL ultrapure water
Sample preparation		
Filtration	with glass fibre filter type APFD 09050<br>(2.7 µm) (Millipore)	with glass fibre filter type APFD 09050 (2.7 µm) (Millipore)
Acidification	Yes, with HCl to pH 3	Yes, with HCl to pH 3
Addition of isotope-labelled internal mixed standard solution (IS)	30 ng EE2-D4, E2-13C2, E1-D4, BPA-D16 and NP-13C6 to each sample	No
Sample enrichment	Solid phase extraction	Solid phase extraction
SPE cartridges	LiChrolut EN RP-18 (bottom: 100 mg LiChrolut EN, top: 200 mg LiChrolut RP 18)	LiChrolut EN RP-18
Conditioning	6 mL hexane<br>2 mL acetone<br>6 mL methanol<br>10 mL water (pH 3.0)	2 mL hexane<br>2 mL acetone<br>6 mL methanol<br>6 mL water (pH 3.0)
Washing	8 mL methanol/water (70:30, v/v)<br>6 mL acetonitrile/water (30:70, v/v)	No, only filling of the cartridge with water (pH 3.0)
Elution	4 mL acetone	4 mL acetone<br>1 mL methanol
Evaporation	With N2 to ca. 100 µL	With N2 to ca. 500 µL, afterwards to 1000 µL with ethanol
Enrichment factor	2500 × wastewater effluent	500 × wastewater effluent<br>1000 × river water
Purification and storage of sample extract		
Sorbent	Mini silica gel columns (1.00 ± 0.01 g)	No
Application of sample	100 µL sample + 2 × 0.2 ml hexane/acetone (60:40, v/v)	
Elution	7.1 mL hexane/acetone (60:40, v/v)	
Evaporation	To dryness, fill-up with 200 µl ethanol	
Storage	In the dark, at -20°C	In the dark, at -20°C

Table E: Specifications of chemical analysis of estrogenic compounds using Liquid Chromatography Mass Spectrometry (LC-MS/MS).

HPLC = High-Performance Liquid Chromathography

**LC-MS/MS analysis**	
LC-MS/MS instrument	API 4000 LC-MS/MS (Applied Biosystems, Warrington, UK)
HPLC separation	Gradient elution<br>Eluent A = water/acetonitrile (90:10, v/v)<br>Eluent B = acetonitrile/water (90:10, v/v)
HPLC column	MS C18 HPLC column (2.1 mm x 100 mm, particle size 3.5 µm)
Ionisation	Negative electrospray ionisation
Calibration	0 - 200 ng/mL E1, E2 and EE2 mixed standards<br>0 - 2500 ng/mL NP+BPA standards (isotope-labelled internal mixed standard solution (IS) added)
Replicates	2
Limit of quantification	estrone 0.6 ng/L; 17β-estradiol 1.1 ng/L; 17α-ethinylestradiol 3.0 ng/L; bisphenol A 4.9 ng/L; nonylphenol 23 ng/L

S4 Material and methods

S4.1 *Gammarus fossarum* feeding activity

Test organism

G. fossarum were sampled using a sieve from an unpolluted tributary of the "Dorfbach" called "Laibrunnenbächli" in a forested area close to Küsnacht, Switzerland (47° 19' 9.16"N, 8° 36' 18.81"E). Subsequent preparation followed Bundschuh et al. [3]. Briefly, gammarids were kept at 13°C for a maximum of one week and divided into three size classes with a passive underwater separation technique [4]. As size, sex and parasitism are known to influence the sensitivity of the test species [5] only male adults (identified by their position in the precopular pair) with a cephalothorax width between 1.2 and 1.6 mm and visually uninfected by parasites were used in the experiments. They were kept in aerated river water from "Laibrunnenbächli" and fed ad libitum with preconditioned black alder leaves (*Alnus glutinosa* L. Gaertn) until the start of the experiments.

Preparation of leaf discs

Leaf disks were prepared as described in Bundschuh et al. [3]. Briefly, leaves of black alder were picked near Landau, Germany (49°11`N; 8°05`E) and stored frozen at -20°C. For further use the leaves were defrosted, cut in discs with 2.0 cm diameter, and conditioned for 10 days in a nutrient medium together with leaves hosting a natural microbial community. Subsequently, the leaf discs were dried at 60°C for 24 h and weighted to the nearest 0.01 mg. Twenty-four hours before the start of the experiment leaves were soaked in water from "Laibrunnenbächli".

Deployment for feeding assay

For an assessment of effects on the feeding activity of *G. fossarum* by wastewater, *in situ* bioassays of 7 days each were performed at a selection of four sampling sites (Buttisholz, Herisau, Hochdorf and Severy) in June 2013. Amphipods were individually exposed in cages together with two preconditioned, weighed and soaked leaf discs, as described in [6]. Each cage (length: 5 cm, diameter: 3 cm) was covered with a 1 mm mesh screen on both sides. Two cage blocks with 10 cages each were deployed at each site with 16 cages containing amphipods and leaf discs and four cages containing leaf discs only to control for microbial and abiotic leaf mass loss over the exposure duration in the absence of amphipods. After an exposure period of 7 days, remaining leaf discs and test species were dried at 60°C for 24 h and weighed to the nearest 0.01 mg.

Data evaluation

Feeding rate of the amphipods was determined as described in [7] and expressed as mg dry leaf material per mg dry weight of *G. fossarum* per day. As not all data were normally distributed, a Mann-Whitney test (GraphPad Prism®, version 5.02 for Windows, GraphPad Software, La Jolla, USA) was performed to assess differences in feeding rates between up- and downstream sites.

S4.2 *Ceriodaphnia dubia* reproduction assay

Test procedure

Daphnids were exposed to dilution series of a reference and samples and effects on mortality and reproduction were assessed over 7 to 8 days. Tests were performed by the private laboratory "Soluval Santiago" (2108 Couvet, Switzerland) according to draft ISO/CD 20665 from 2005 [8] and AFNOR T90-376 [9]. The test was carried out with a slightly modified version of the standards: the dilution medium corresponded to a moderately hard water prepared by mixing 25% of Evian mineral water, 25% of Elendt M4 medium [10] and 50% of deionised water, supplemented with selenium and vitamin B12. Food consisted of a mixture of yeast, digested fish flake suspension (TetraMin®) and green algae (*R. subcapitata* and *Chlorella* sp.). Neonates that were less than 24 h old, and within 8 h of the same age, were exposed for up to 8 days to a single concentration (90%) of the environmental samples in a static-renewal system (12 replicates per concentration). Control water (i.e. dilution medium) was tested using 20 replicates. All tests were carried out at 25 ± 1°C in a temperature controlled chamber; illumination ranged from 300 to 500 lux, with a light-dark period of 16:8 h. Water was renewed every day, except for day 1. On day 1 and each following day at the time of water renewal, survival of mothers was determined and offspring were counted. Physicochemical

characteristics of the sample solutions (pH, dissolved oxygen [mg/L] and conductivity [µS/cm]) were measured during the test in regular intervals (n = 5-6).

Data evaluation

As not all data were normally distributed, a Mann-Whitney test (GraphPad Prism®, version 5.02 for Windows) was performed to assess differences in number of offspring between up- and downstream sites.

S4.3 ERα-CALUX®

The ERα-CALUX® with the U2OS human cell line with a luciferase gene under the transcriptional control of responsive elements for activated hormone receptors was performed according to Van der Linden, Heringa [11]. In short, cells were seeded into 96-well plates with DF medium (without phenol red) that was supplemented with stripped (dextran-coated charcoal treated) serum. After 24 h of incubation (37 °C, 5.0% CO2), the medium was replaced by medium containing the water extracts (0.1 - 0.5% DMSO) for agonistic activity testing. After 24 h of incubation, the medium was removed and the cells were lysed in 30 µL of Triton-lysis buffer. The amount of luciferase activity was quantified using a luminometer (MicroLumat Plus, Berthold Technologies, Switzerland). On all plates, a dose-response curve of the reference compound 17β-estradiol was included for quantification of the response as well as a solvent control (DMSO, 0.1%/well, n = 3 wells/plate). Both, the reference substance and the water sample extracts, were assessed in triplicate in a dilution series, with the initial concentration of 17β-estradiol being 1.0 x 10^{-10} M and maximum enrichment factors of the wastewater samples of 5 (WWTP effluent) and 8.6 - 50 (river water). Only dilutions that showed no cytotoxicity (based on the microscopic evaluation of the cell viability) were used for quantification of the response.

S5 Equations for bioassay data analysis

$$Induction = Bottom + \frac{(Top - Bottom)}{1 + 10^{(LogEC50 - X) * Hill\ Slope}} \quad (1)$$

With

X = Log of dose or concentration

Top = Maximum response (fitted for the reference curve)

Bottom = Minimum response (fixed to the measurement of the solvent blank)

$LogEC_{50}$ = Log of concentration at which 50% of the maximum response is observed

HillSlope = Slope factor

Induction data of the reference and test sample were then normalised using Equation 2, where response refers to the pertinent measured activity in the assay.

$$Induction\ [\%] = \frac{Response - Bottom}{Top - Bottom} \quad (2)$$

The estrogenic activity of the sample (EEQ_{sample}) was determined by dividing the concentration of the positive control (PC) needed for 10% effect (PC_{10}) by the relative enrichment factor (REF) necessary to produce 10% effect (REF_{10}) (Equation 3).

$$EEQ_{sample} = PC_{10}/REF_{10} \quad (3)$$

S6 Relative effect potencies for the *in vitro* bioassays

Relative effect potencies for the measured estrogenic compounds for the YES and the ERα-CALUX® are listed in Table F.

Table F: 17β-estradiol equivalence factors (EEF) for estrone (E1), 17β-estradiol (E2), 17α-ethinylestradiol, bisphenol A und nonylphenol. Literature values.

Compound name	EEF for Yeast Estrogen Screen[1, 2]	EEF for ERα-CALUX® [2, 3]
Estrone	0.26	0.02
17β-estradiol	1.0	1.0
17α-ethinylestradiol	1.2	1.3
Bisphenol A	6.5×10^{-5}	2.7×10^{-5}
Nonylphenol	2.5×10^{-5}	2.4×10^{-5}

[1][12], [2][13] [3][14]

Table G gives an overview on relative effect potencies for photosystem II inhibitors in the combined algae assay.

Table G: Diuron equivalence factors (DEF) for photosystem II inhibiting herbicides in the combined algae assay.

Mean values of three experiments from 2014, 2016 and 2017 at the Ecotox Centre, n = 2 technical and 3 biological replicates per experiment. DEF were calculated individually for each experiment and the average of these three experiments reported.

Compound name	Use class	EC_{50}	DEF
Atrazine	Pesticide	2.96×10^{-7}	0.065
Bentazon	Pesticide	1.75×10^{-4}	0.000
Chloridazon	Pesticide	3.15×10^{-6}	0.007
Chlortoluron	Pesticide	8.19×10^{-8}	0.201
Dimefuron	Pesticide	9.90×10^{-8}	0.168
Diuron	Biocide	1.74×10^{-8}	1
Isoproturon	Pesticide	9.99×10^{-8}	0.175
Lenacil	Pesticide	2.44×10^{-8}	0.691
Linuron	Pesticide	4.51×10^{-8}	0.366
Metamitron	Pesticide	1.04×10^{-6}	0.016
Metribuzin	Pesticide	4.18×10^{-8}	0.395
Monolinuron	Pesticide	6.23×10^{-7}	0.027
Monuron	Pesticide	2.18×10^{-7}	0.076
Prometryn/Terbutryn	Biocide	2.11×10^{-8}	0.874*
Propazin-2-hydroxy / Terbuthylazin-2-hydroxy	Pesticide (metabolite of terbuthylazine)		0.337**
Simazine	Pesticide	3.38×10^{-7}	0.050
Terbutryn	Biocide	2.11×10^{-8}	0.874
Terbuthylazine	Pesticide	5.17×10^{-8}	0.337

* relative potency for terbutryn applied
** relative potency from parent compound terbuthylazine applied

Table H lists relative effect potencies for organophosphate and carbamate insecticides active in the acetylcholinesterase inhibition assay.

Table H: Parathion equivalence factors (PtEF) for acetylcholinesterase inhibiting insecticides in the acetylcholinesterase inhibition assay.

Mean values from three test runs (biological replicates) conducted in 2018 at the Ecotox Centre, n = 2 technical replicates per test run.

Compound name	Insecticide classification[1]	EC_{50}	PtEF
Aldicarb	oxime carbamate insecticide	4.73×10^{-8}	7.30
Azamethiphos	heterocyclic organothiophosphate insecticide[2]	5.51×10^{-8}	6.26
Carbofuran	benzofuranyl methylcarbamate insecticide	3.28×10^{-8}	10.52
Chlorfenvinphos	organophosphate insecticide	2.55×10^{-6}	0.14
Chlorpyrifos	pyridine organothiophosphate insecticide[2]	3.59×10^{-7}	0.96
Chlorpyrifos-methyl	pyridine organothiophosphate insecticide[2]	7.34×10^{-7}	0.47
Diazinon	pyrimidine organothiophosphate insecticide[2]	5.63×10^{-6}	0.06
Dichlorvos	organophosphate insecticide	1.16×10^{-6}	0.30
Methiocarb	phenyl methylcarbamate insecticide	8.35×10^{-7}	0.41
Methiocarb-sulfoxide		4.64×10^{-6}	0.07
Methomyl	oxime carbamate insecticide	3.26×10^{-7}	1.06
Paraoxon	oxidized form of parathion	1.76×10^{-7}	1.96
Parathion	phenyl organothiophosphate insecticide[2]	3.45×10^{-7}	1.00
Pirimicarb	dimethylcarbamate insecticides	8.44×10^{-5}	4.08×10^{-3}

[1] http://www.alanwood.net/pesticides/class_insecticides.html
[2] only active after metabolic oxidation

S7 Applied annual average environmental quality standards (AA-EQS) for risk assessment based on individual chemicals

Table I: Annual average environmental quality standards (AA-EQS, ng/L) for the individual estrogenic compounds.

Compound name	CAS-Nr	AA-EQS (ng/L)	Reference
Estrone (E1)	53-16-7	3.6	1
17β-estradiol (E2)	50-28-2	0.4	1
17α-ethinylestradiol (EE2)	57-63-6	0.037	1
Nonylphenol (NP)	104-40-5	43	1
Bisphenol A (BPA)	80-05-7	240	1

1 - OZ (http://www.oekotoxzentrum.ch/expertenservice/qualitaetskriterien/qualitaetskriterienvorschlaege-oekotoxzentrum/)

Table J: Annual average environmental quality standards (AA-EQS, ng/L) for the individual photosystem II-inhibitors (adapted from [15]).

Compound name	CAS-Nr	AA-EQS (ng/L)	Reference
Atrazine	1912-24-9	600	1
Bentazon	25057-89-0	270000	2
Chloridazon	1698-60-8	10000	2
Chlortoluron	15545-48-9	600	1
Dimefuron	34205-21-5	8	3
Diuron	330-54-1	70	2
Isoproturon	34123-59-6	640	2
Lenacil	2164-08-1	1000	4
Linuron	330-55-2	260	2
Metamitron	41394-05-2	4000	2
Metribuzin	21087-64-9	58	2
Monolinuron	1746-81-2	150	5
Monuron	150-68-5	200	6
Prometryn/Terbutryn	886-50-0	65*	2
Propazin-2-hydroxy/Terbutylazine-2-hydroxy	66753-07-9	220**	2
Simazine	122-34-9	1000	1
Terburtryn	886-50-0	65	2
Terbuthylazine	5915-41-3	220	2

1 - EU WFD 2013; list of priority substances (http://eur-lex.europa.eu/LexUriServ/LexUriServ.do?uri=CELEX:32008L0105:EN:NOT)
2 - OZ (http://www.oekotoxzentrum.ch/expertenservice/qualitaetskriterien/qualitaetskriterienvorschlaege-oekotoxzentrum/)
3 - OZ ad hoc
4 - UBA (Jahnel et al. 2006)
5 - RIVM (http://www.rivm.nl/bibliotheek/rapporten/601716009.pdf)
6 - RIVM ad hoc (http://www.rivm.nl/rvs/dsresource?type=pdf&objectid=rivmp:290942&type=org&disposition=inline)
* AA-EQS for terbutryn applied
** AA-EQS from main compound terbuthylazine applied

Table K: Annual average environmental quality standards (AA-EQS, ng/L) for the acetylcholinesterase inhibitors (adapted from [15]).

Compound name	CAS-Nr	AA-EQS (ng/L)	Reference
Aldicarb	116-06-3	100	1
Azamethiphos	35575-96-3	20	2
Carbofuran	1563-66-2	16	3
Chlorfenvinphos	470-90-6	100	4
Chlorpyrifos	2921-88-2	0.46	4
Chlorpyrifos-methyl	5598-13-0	0.2	5
Diazinon	333-41-5	12	6
Dichlorvos	62-73-7	0.6	7
Dimethoate	60-51-5	70	6
Fenoxycarb	72490-01-8	0.3	8
Methiocarb	2032-65-7	2	9
Methiocarb-sulfoxide	2635-10-1	56	10
Methomyl	16752-77-5	160	3
Pirimicarb	23103-98-2	90	6

1 - http://www.rivm.nl/rvs/Normen/Eindresultaat?groep=normen&waarde=aldicarb&lijst=milieukwaliteit&veld=substancename_tagged
2 - http://www.sepa.org.uk/water/water_regulation/guidance/pollution_control.aspx
3 - OZ ad hoc
4 - EU WFD 2013, list of priority substances (http://eur-lex.europa.eu/LexUriServ/LexUriServ.do?uri=CELEX:32008L0105:EN:NOT)
5 - RIVM ad hoc (https://rvs.rivm.nl/zoeksysteem?groep=normen&waarde=5598-13-0&lijst=milieukwaliteit&veld=casnumber_tagged)
6 - OZ (http://www.oekotoxzentrum.ch/expertenservice/qualitaetskriterien/qualitaetskriterienvorschlaege-oekotoxzentrum/)
7 - EU WFD proposal 2012
8 - RIVM-Bericht 2008 (https://www.rivm.nl/bibliotheek/rapporten/601716008.pdf)
9 - RIVM (Ctgb) (http://www.rivm.nl/rvs/Normen/Eindresultaat?groep=normen&waarde=2032-65-7&lijst=milieukwaliteit&veld=casnumber_tagged)
10 - http://www.bioforsk.no/ikbViewer/page/prosjekt/tema/artikkel?p_dimension_id=18844&p_menu_id=18851&p_sub_id=18845&p_document_id=91227&p_dim2=18854

S8 Results

S8.1 General water chemistry

Results for general water quality parameters are presented in [2] and briefly recapitulated here. Nutrient concentrations generally increased from up- to downstream sites. These differences were most pronounced for phosphate (PO_4^{3-}), total phosphorus (TP) and ammonium (NH_4^+) with mean concentrations (+/- SD) of 16.2 ± 12 µg P L^{-1} (upstream) and 58.5 ± 36.6 µg P L^{-1} (downstream), 37.7 ± 21.1 µg P L^{-1} (upstream) and 100.3 ± 2.4 µg P L^{-1} (downstream) and from 23.8 ± 14.0 µg N L^{-1} to 86.6 ± 123.6 µg N L^{-1} for PO_4^{3-}, TP, and NH_4^+, respectively with a substantial variation among sites. Dissolved organic carbon (DOC) and nitrate (NO_3^-) values were similar up- and downstream with values increasing from 2.5 ± 0.9 mg DOC L^{-1} to 3.0 ± 0.9 mg DOC L^{-1} and from 3.2 ± 1.8 mg N L^{-1} to 4.6 ± 2.0 mg N L^{-1} for DOC and NO_3^- respectively.

As reported by Burdon, Reyes [2], water quality at upstream sites indicated an influence of the catchment with e.g. a majority of streams being classified as ‘moderate’ to ‘very bad’ with regard to phosphorus concentrations according to Swiss assessment protocols [16]. Nitrate and dissolved phosphorous were also correlated with agricultural parameters such as arable cropping or cover of cropping and pasture in the catchment. Further details on these correlations can be found in [2].

S8.2 Micropollutant screening

Tables L, M, and N give an overview on the results from measurements of detected estrogenic compounds, photosystem II and acetylcholinesterase inhibitors.

Table L: Summary of results from estrogen measurements (ng/L).

Data are provided in S1_Data. 17α-ethinylestradiol was always below LOQ. EEQ_{chem} = estradiol equivalent concentration based on results of chemical analysis.

	EEQ_{Chem}	Estrone	17β-Estradiol	Bisphenol A	Nonylphenol
Number of values	24	24	10	24	12
Minimum	0.042	0.16	0.25	3.7	7.2
25% Percentile	0.18	0.49	0.28	11	49
Median	0.43	1.3	0.41	22	101
75% Percentile	0.95	2.2	0.83	78	128
Maximum	6.8	19	1.8	257	218
Mean	0.85	2.3	0.61	47	102
Std. Deviation	1.4	3.8	0.49	56	65
Std. Error	0.28	0.79	0.15	11	19
Lower 95% CI of mean	0.26	0.63	0.26	23	61
Upper 95% CI of mean	1.4	3.9	0.96	71	143
Coefficient of variation	164%	171%	79%	119%	63%
Sum	20	54	6.1	1133	1227

Table M: Summary of results from measurements of photosystem II inhibitors (ng/L)

Data are provided in the SI of Munz, Burdon [1]. WWTP = wastewater treatment plant, US = upstream, DS = downstream

	Atrazine			Bentazone		Chloridazone		Chlortoluron			Dimefuron		Diuron			Isoproturone			Lenacil		Linuron	
	WWTP	US	DS	US	DS	US	DS	WWTP	US	DS	US	DS	WWTP	US	DS	WWTP	US	DS	US	DS	US	DS
Number of values	63	89	89	13	13	7	9	10	27	30	1	1	80	68	91	53	56	71	6	6	2	5
Minimum	2.9	0.6	0.6	2.7	2.2	1.4	0.5	2	0.8	0.8	9.2	8.2	7.9	0.5	0.8	1.6	0.4	0.3	7.9	8.8	1.7	1.7
25% Percentile	5.8	4.1	4	6.2	6.8	5.2	1.8	5	1.9	1.9	9.2	8.2	23	1.7	4.5	3.9	0.83	1.6	9.5	13	1.7	1.8
Median	9.2	5.4	5.9	13	19	13	6.3	10	3.1	2.5	9.2	8.2	40	3.1	9.7	11	1.6	2.7	13	15	2	3.2
75% Percentile	15	8	8.8	70	68	18	19	15	8	6.9	9.2	8.2	82	6.6	20	24	3.3	7.7	28	31	2.2	57
Maximum	420	17	99	410	330	34	33	27	26	25	9.2	8.2	960	54	220	260	57	63	66	67	2.2	110
Mean	22	6.1	8.7	71	69	13	11	11	6.3	5.4	9.2	8.2	97	6.4	22	22	4.1	6.5	21	23	2	24
Std. Deviation	58	3.2	12	125	114	11	11	8.4	7.4	6.3	0	0	179	8.8	37	40	8.2	11	22	22	0.35	48
Std. Error	7.3	0.34	1.2	35	32	4.1	3.7	2.7	1.4	1.2	0	0	20	1.1	3.9	5.5	1.1	1.3	9.1	8.9	0.25	21
Lower 95% CI of mean	7.8	5.5	6.2	-4.8	0.28	3.4	2.9	5.1	3.4	3	0	0	57	4.3	14	11	1.9	4	-2.6	0.31	-1.2	-36
Upper 95% CI of mean	37	6.8	11	146	138	24	20	17	9.2	7.8	0	0	137	8.5	30	33	6.3	9.1	44	46	5.1	84
Coefficient of variation	259%	52%	135%	177%	165%	81%	97%	76%	118%	118%	0%	0%	184%	137%	169%	180%	198%	164%	107%	94%	18%	199%
Sum	1403	547	773	920	897	94	102	111	170	162	9.2	8.2	7776	435	2009	1179	232	464	125	139	3.9	121

Table M continued: Summary of results from measurements of photosystem II inhibitors (ng/L).

Data are provided in the SI of Munz, Burdon [1]. WWTP = wastewater treatment plant, US = upstream, DS = downstream

	Metamitron		Metribuzin			Monolinuron		Monuron		Prometryn/ Terbutryn		Propazin-2-Hydroxy / Terbuthylazine-2-Hydroxy		Simazine			Terbutryn			Terbuthylazine		
	US	DS	WWTP	US	DS	US	DS	US	DS	US	DS	US	DS	WWTP	US	DS	WWTP	US	DS	WWTP	US	DS
Number of values	10	12	13	19	27	1	1	1	1	7	6	23	24	38	72	74	71	34	68	66	88	87
Minimum	2.9	2.8	1.7	0.5	0.4	6	4.9	5.6	9.8	0.2	0.5	1.2	1.6	2.4	0.5	0.7	2.7	0.6	0.8	2	1	0.9
25% Percentile	3.4	3.2	6.7	1.5	1.5	6	4.9	5.6	9.8	0.3	0.73	4.1	5.8	3.9	1	1.4	7.5	0.9	1.9	5.2	2.7	3.5
Median	12	8.6	8.7	2.3	2.1	6	4.9	5.6	9.8	0.5	1.6	7.1	9	5.1	1.3	1.8	12	1.2	3.3	8.1	4.2	6.1
75% Percentile	44	48	18	16	16	6	4.9	5.6	9.8	2.9	5	13	13	7.9	1.7	2.6	27	1.8	6.1	19	8.5	14
Maximum	79	210	720	58	330	6	4.9	5.6	9.8	3.3	7	22	95	52	4.7	39	690	16	110	630	230	3000
Mean	23	37	74	9	18	6	4.9	5.6	9.8	1.4	2.6	8.3	13	10	1.5	3	28	2.1	7	28	15	54
Std. Deviation	26	61	198	14	63	0	0	0	0	1.3	2.5	5.3	18	12	0.83	4.8	81	2.8	14	80	37	323
Std. Error	8.2	17	55	3.3	12	0	0	0	0	0.5	1	1.1	3.8	2	0.1	0.56	9.7	0.48	1.7	9.8	3.9	35
Lower 95% CI of mean	5	-1.7	-45	2.1	-6.7	0	0	0	0	0.14	-0.04	6.1	5.4	6.1	1.3	1.9	9.1	1.1	3.6	8.2	7.4	-15
Upper 95% CI of mean	42	75	193	16	43	0	0	0	0	2.6	5.3	11	21	14	1.7	4.1	48	3.1	10	47	23	123
Coefficient of variation	110%	165%	267%	159%	346%	0%	0%	0%	0%	97%	97%	63%	139%	120%	56%	160%	287%	133%	199%	287%	243%	599%
Sum	235	441	961	170	491	6	4.9	5.6	9.8	9.6	16	192	317	381	108	224	2013	72	478	1835	1338	4688

Table N: Summary of results from measurements of acetylcholinesterase inhibitors (ng/L).

Data are provided in the SI of Munz, Burdon [1]. WWTP = wastewater treatment plant, US = upstream, DS = downstream

	Carbofuran		Chlorpyrifos		Chlorpyrifos-Methyl		Diazinon			Dimethoate			Fenoxycarb	Methiocarb	Pirimicarb	
	US	DS	US	DS	WWTP	DS	WWTP	US	DS	WWTP	US	DS	DS	DS	WWTP	DS
Number of values	1	1	1	1	1	1	74	12	67	8	5	9	2	1	4	5
Minimum	1.5	7.7	3.9	5.2	73	15	2.1	0.4	0.5	2.1	0.5	0.6	3	0.8	13	2.2
25% Percentile	1.5	7.7	3.9	5.2	73	15	4.9	0.5	0.9	2.4	0.85	1.1	3	0.8	18	3.1
Median	1.5	7.7	3.9	5.2	73	15	7.4	1	2.2	4.1	2.1	2.2	4.3	0.8	37	3.9
75% Percentile	1.5	7.7	3.9	5.2	73	15	13	1.9	5.7	101	3.3	27	5.5	0.8	235	36
Maximum	1.5	7.7	3.9	5.2	73	15	190	18	130	440	4.2	97	5.5	0.8	300	63
Mean	1.5	7.7	3.9	5.2	73	15	18	2.5	7.2	77	2.1	18	4.3	0.8	97	16
Std. Deviation	0	0	0	0	0	0	31	4.9	17	152	1.4	32	1.8	0	136	26
Std. Error	0	0	0	0	0	0	3.6	1.4	2.1	54	0.63	11	1.3	0	68	12
Lower 95% CI of mean	0	0	0	0	0	0	11	-0.68	2.9	-50	0.34	-6.9	-12	0	-120	-16
Upper 95% CI of mean	0	0	0	0	0	0	25	5.6	11	204	3.8	43	20	0	313	49
Coefficient of variation	0%	0%	0%	0%	0%	0%	174%	201%	241%	197%	67%	180%	42%	0%	141%	161%
Sum	1.5	7.7	3.9	5.2	73	15	1316	29	479	617	10	162	8.5	0.8	387	81

S8.3 Results of *in vitro* bioassays

Tables O, P, and Q summarize the results of the YES, the ERα-CALUX®, the combined algae assay and the AChE inhibition assay.

Table O: Summary of results from the Yeast Estrogen Screen (YES) and the ERα-CALUX®.

EEQ_{bio} = 17β-estradiol equivalent, WWTP = wastewater treatment plant, US = upstream, DS = downstream. Data are provided in S2_Data and S3_Data.

	$EEQ_{bio, YES}$ (ng/L)			$EEQ_{bio, ERα-CALUX®}$ (ng/L)		
	WWTP	US	DS	WWTP	US	DS
Number of values	24	22	24	12	11	12
Minimum	0.11	0.04	0.05	0.53	0.06	0.09
25% Percentile	0.46	0.05	0.10	0.71	0.06	0.16
Median	0.63	0.07	0.19	0.99	0.08	0.30
75% Percentile	0.96	0.11	0.26	2.00	0.12	0.49
Maximum	3.60	0.27	0.85	3.60	0.37	0.84
Mean	0.83	0.08	0.22	1.40	0.11	0.34
Std. Deviation	0.74	0.06	0.17	0.98	0.09	0.23
Std. Error	0.15	0.01	0.03	0.28	0.03	0.07
Lower 95% CI of mean	0.52	0.06	0.14	0.79	0.06	0.19
Upper 95% CI of mean	1.10	0.11	0.29	2.00	0.17	0.48
Coefficient of variation	88%	66%	78%	69%	78%	67%
Sum	20.00	1.90	5.20	17.00	1.30	4.10

Table P: Summary of results from the combined algae assay.

DEQ_{bio} = diuron equivalent concentration based on bioassay results, WWTP = wastewater treatment plant, US = upstream, DS = downstream. Data are provided in S4_Data.

	PSII inhibition						Growth inhibition		
	DEQ_{bio} (ng/L, 2h)			DEQ_{bio} (ng/L, 24h)			DEQ_{bio} (ng/L, 24h)		
	WWTP	US	DS	WWTP	US	DS	WWTP	US	DS
Number of values	24	24	24	24	24	24	24	24	24
Minimum	27.2	2.4	2.8	37.8	4.2	5.2	300.5	59.3	100.8
25% Percentile	63.6	6.5	19.1	143.3	10.9	31.2	784.1	111.8	212.9
Median	126.1	24.5	42.7	299.8	29.5	67.1	1233.0	185.4	340.8
75% Percentile	278.0	41.7	91.8	467.2	66.7	183.2	1876.0	381.9	614.3
Maximum	791.0	122.4	1576.0	981.7	180.5	2460.0	2828.0	1203.0	3845.0
Mean	187.2	33.0	125.6	350.7	44.2	193.9	1370.0	283.7	558.6
Std. Deviation	183.5	33.0	314.9	235.3	42.8	489.4	682.5	257.0	752.5
Std. Error	37.5	6.7	64.3	48.0	8.7	99.9	139.3	52.5	153.6
Lower 95% CI of mean	109.7	19.1	-7.4	251.4	26.1	-12.7	1082.0	175.2	240.8
Upper 95% CI of mean	264.7	46.9	258.6	450.1	62.3	400.6	1658.0	392.2	876.4
Coefficient of variation	98%	100%	251%	67%	97%	252%	50%	91%	135%
Sum	4493	792.1	3015	8418	1060	4654	32888	6808	13407

Table Q: Summary of results from the acetylcholinesterase inhibition assay.

$PtEQ_{bio}$ = parathion equivalent concentration based on bioassay results, WWTP = wastewater treatment plant, US = upstream, DS = downstream. Data are provided in S6_Data.

	$PtEQ_{bio}$ (ng/L)		
	**WWTP**	**US**	**DS**
Number of values	24	24	24
Minimum	633.4	37.12	71.63
25% Percentile	967.3	126.9	175.8
Median	1173	247.7	405.3
75% Percentile	1477	341.2	567
Maximum	2183	534.4	1278
Mean	1258	258.5	411.1
Std. Deviation	392.6	143.4	269.3
Std. Error	80.14	29.26	54.98
Lower 95% CI of mean	1092	198	297.4
Upper 95% CI of mean	1423	319.1	524.8
Coefficient of variation	31%	55%	66%
Sum	30184	6204	9866

S8.4 Results of ERα-CALUX®

Estrogenic activity in ERα-CALUX®, which was quantified at 12 sites in 2014, was similar to the one measured with YES, both values were significantly correlated, however, ERα-CALUX® generally resulted in higher EEQ_{bio} values than YES (Table O and Fig C).

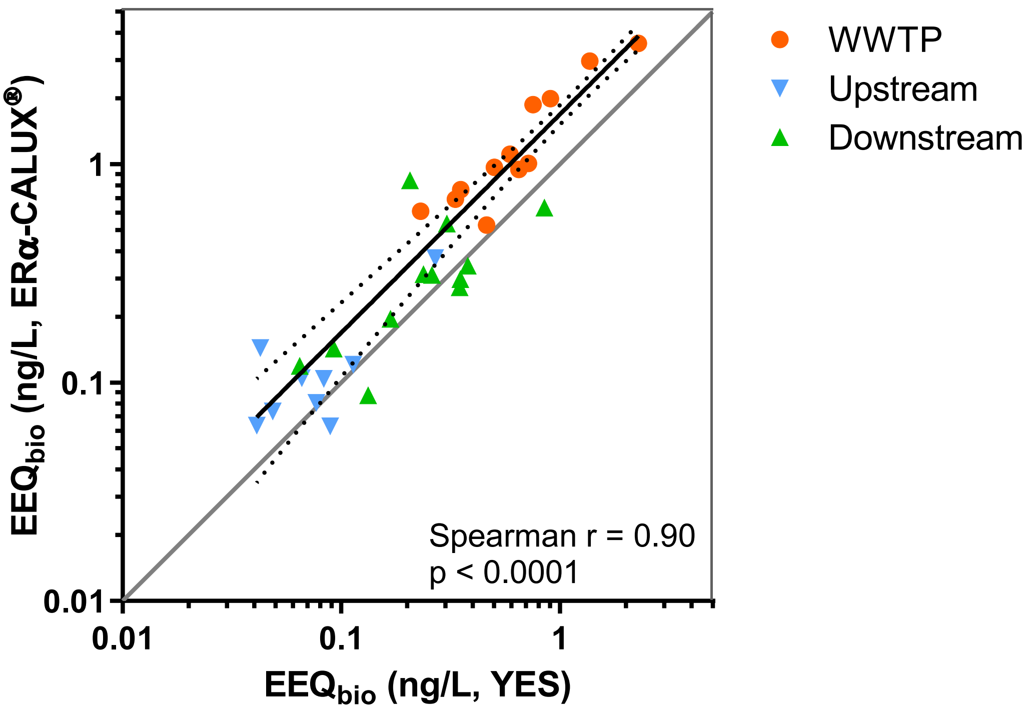

Fig C: Correlation of 17β-estradiol equivalent concentrations (EEQ_{bio}, ng/L) in the Yeast Estrogen Screen (YES) and the ERα-CALUX® at 12 sites investigated in 2014 in wastewater treatment plant (WWTP) effluent as well as in the river up- and downstream of the WWTP discharge.

Mean values, n = 32. Correlation: all data $p < 0.0001$, WWTP $p < 0.0001$, Upstream $p > 0.05$, downstream $p < 0.0220$. The black line shows the non-linear regression ± 95% confidence interval. The grey line indicates the 1:1 line.

Mean EEQ values were lowest at the upstream sites with 0.12 ng/L and increased significantly to the downstream sites. As with the YES, significantly higher mean EEQ values were detected in the WWTP effluent compared to the river (1.42 ng/L) (Table O and Fig D**Error! Reference source not found.**). Values varied in the same range as for the YES (coefficient of variation: 66 - 77 %).

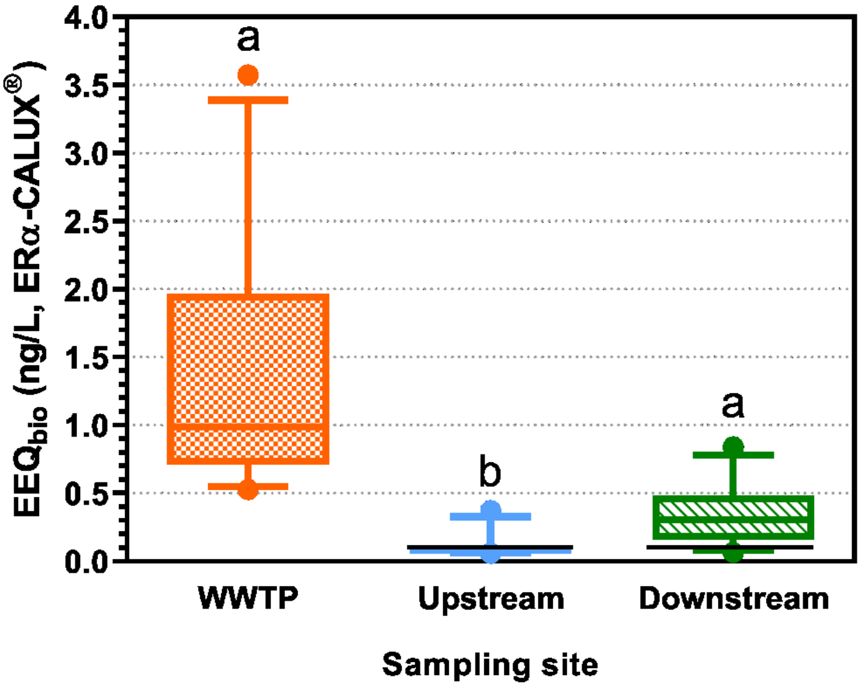

Fig D: Estrogenic activity in the ERα-CALUX®: 17β-estradiol equivalent concentrations (EEQ_{bio}, ng/L) at 12 sites investigated in 2014 in wastewater treatment plant (WWTP) effluent as well as in the river up- and downstream of the WWTP discharge.

Box-Whisker plots with the line representing the median, the box the mean 50% of the data and the Whiskers the 10-90 percentile. Dots represent values outside this range. n = 12. Different letters indicate significant differences (Friedman test with Dunn's Multiple Comparison Test). Limits of quantification were determined for each sample and ranged from 0.01-0.05 ng EEQ/L for WWTP effluent and 0.01 - 0.03 ng EEQ/L for river samples. The black line represents the effect-based trigger value (0.1 ng/L) [17].

EEQ_{bio} values from ERα-CALUX® were significantly correlated to EEQ_{chem} values (Fig E). However, the correlation was less strong than for the YES, but it has to be kept in mind that only values from 12 sites were available for ERα-CALUX® whereas values from 24 sites could be used for YES.

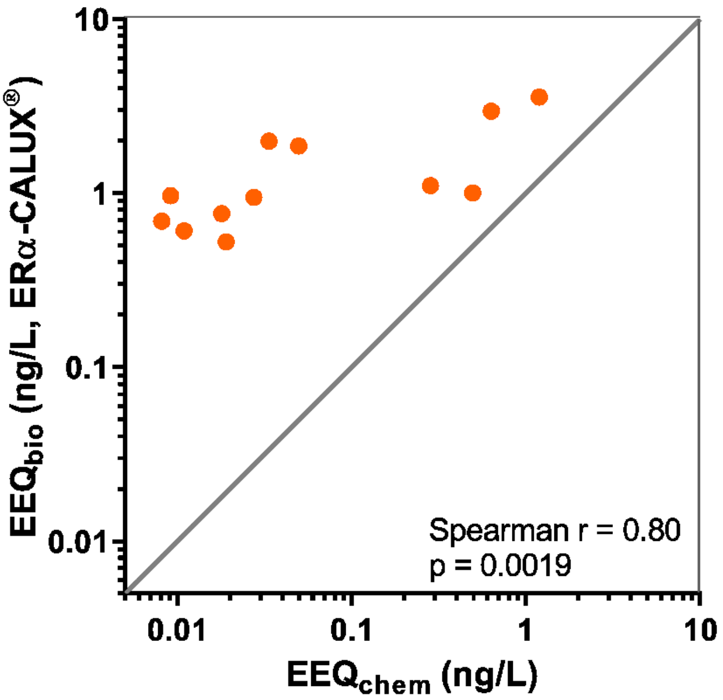

Fig E: Correlation of 17β-estradiol equivalent concentrations measured in the ERα-CALUX® (EEQ_{bio}, ng/L) to the values calculated by chemical analysis (EEQ_{chem}, ng/L) based on relative potencies of the measured estrogens in the bioassay.

EEQs from 12 sites investigated in 2014 in wastewater treatment plant effluent. The grey line indicates the 1:1 line.

For the ERα-CALUX® a lower percentage of the EEQ_{bio} values could be explained by EEQ_{chem} as for the YES, in part also due to the generally higher EEQ_{bio} values in this assay. Over all sites in the mean 12% of the observed effects could be explained by the measured estrogens, with E1 contributing 2%, E2 10%, BPA 0.1% and NP 0.3%. If chemicals below LOQ would be included as half of the LOQ value in the calculations, about 40% of the observed estrogenic activity could be explained, with E2 and EE2 being the most important contributors to this activity (explaining 18 and 19% respectively) (Fig F). The differences in % contribution of the different estrogens for YES and ERα-CALUX® are also due to differences in the relative potencies for the individual estrogens in both bioassays (Table F).

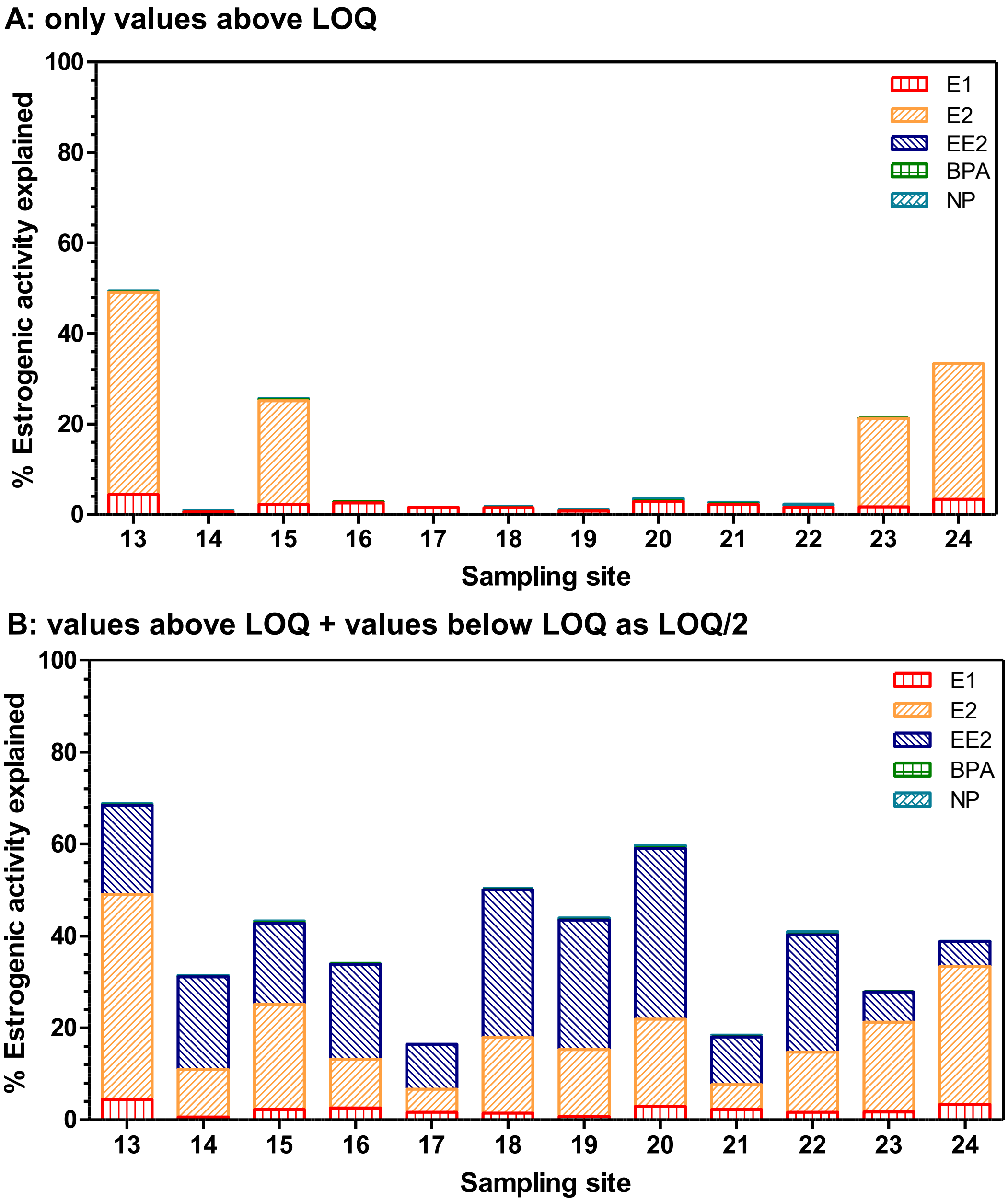

Fig F: Contribution of individual estrogenic compounds measured in chemical analysis (EEQ_{chem}, based on relative potencies of the measured estrogenic compounds in the bioassay) to the 17β-estradiol equivalent concentrations (EEQ_{bio}, ng/L) measured in the ERα-CALUX®.

Mean values from wastewater treatment plant effluent of 12 sites investigated in 2014. A and B represent different options of integration of the values below LOQ. E1 = estrone, E2 = 17β-estradiol, EE2 = 17α-ethinylestradiol, BPA = bisphenol A, NP = nonylphenol. LOQs were 0.1 ng/L (E1), 0.2 ng/L (E2), 0.3 ng/L (EE2), 1.6 ng/L (BPA) and 1.2 ng/L (NP).

S8.5 *In vitro* bioassays and dissolved organic carbon

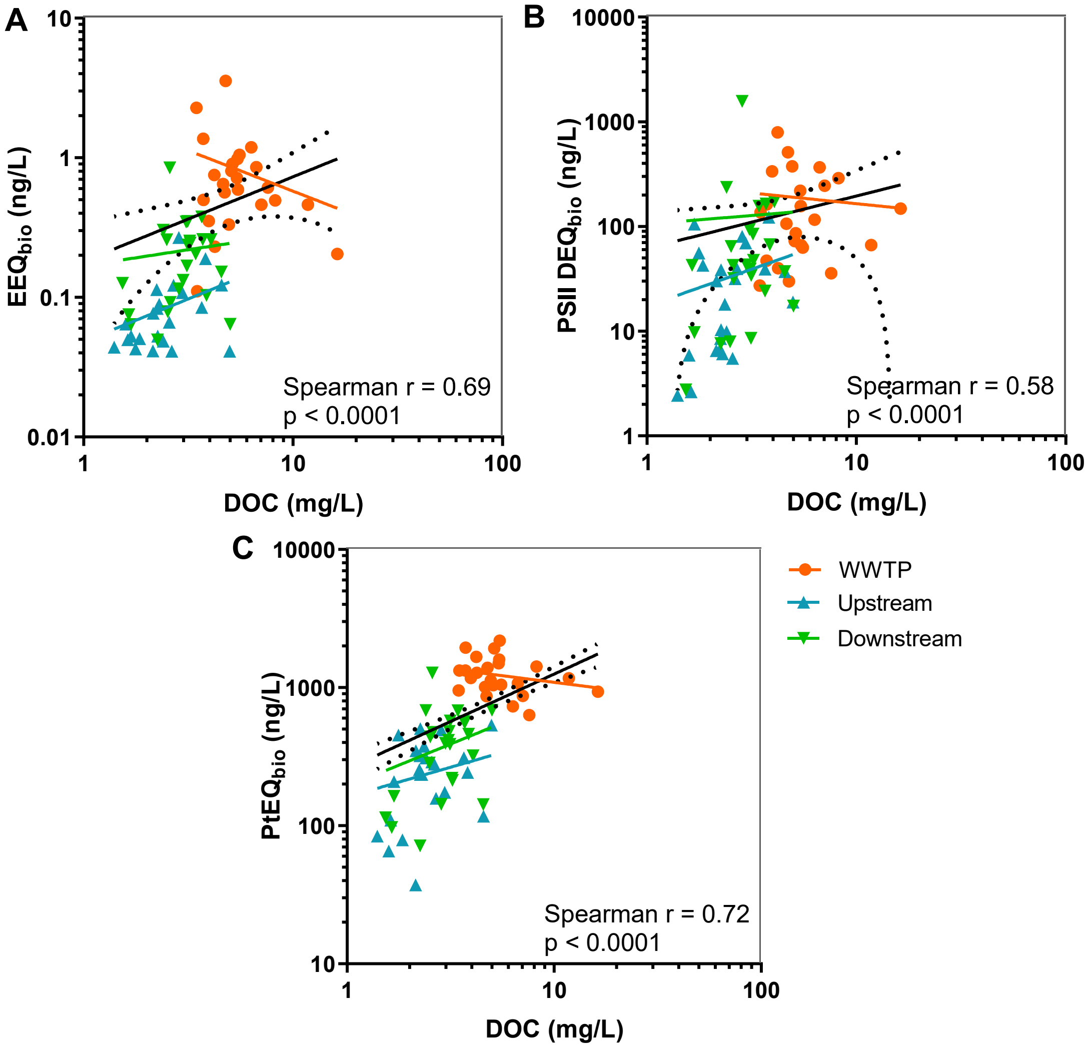

Fig G: Correlation of (A) 17β-estradiol equivalent concentrations (EEQ_{bio}, ng/L) measured in the Yeast Estrogen Screeen, (B) diuron equivalent concentrations (DEQ_{bio}, ng/L) measured in the combined algae assay, and (C) parathion equivalent concentrations ($PtEQ_{bio}$, ng/L) measured in the acetylcholinesterase inhibition assay to dissolved organic carbon (DOC) values (mg/L) measured in the water samples.

Values from wastewater treatment plant effluent (orange dots), and river water upstream (blue triangles) and downstream (green triangles) of the WWTP effluent discharge at 24 sites investigated in 2013 and 2014 (n = 72). Black lines indicate the overall non-linear regression ± 95% confidence interval, orange, blue and green lines show the non-linear regression for WWTP effluent (WWTP), upstream (US), and downstream (DS) sites respectively.

S8.6 Adding compounds below LOQ as LOQ/2

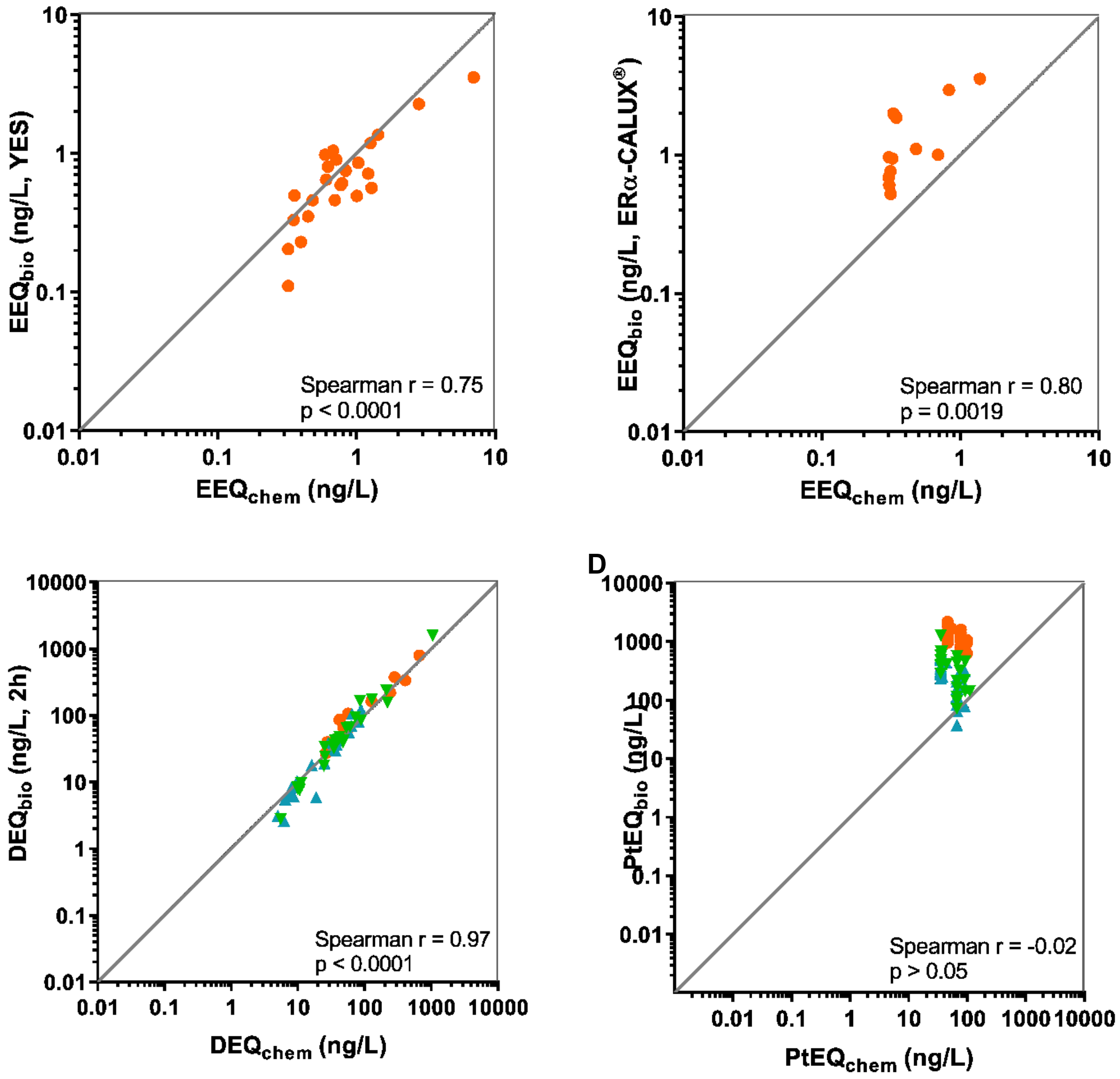

Fig H: Correlation of bioanalytical equivalent concentrations measured in (A) the Yeast Estrogen Screen (YES, 17β-estradiol equivalent concentrations, EEQ$_{bio}$, ng/L), (B) the ERα-CALUX® (EEQ$_{bio}$, ng/L), (C) the combined algae assay (diuron equivalent concentrations, DEQ$_{bio}$, ng/L), and (D) the acetylcholinesterase (AChE) inhibition assay (parathion equivalent concentrations, PtEQ$_{bio}$, ng/L) to the values calculated by chemical analysis (EEQ$_{chem}$, DEQ$_{chem}$, PtEQ$_{chem}$, resp.) based on relative potencies of the measured estrogens, PSII inhibitors or AChE inhibitors in the bioassays. Values below LOQ were included as LOQ/2.

BEQs at 24 sites investigated in 2013/2014 in wastewater treatment plant effluent (orange dots; all bioassays) as well as in the river upstream (blue triangles) and downstream (green triangles) of the WWTP discharge (combined algae assay and AChE inhibition assay). Mean. N = 24 (YES), n = 12 (ERα-CALUX®), n = 60 (algae, AChE). Grey lines indicate the 1:1 line.

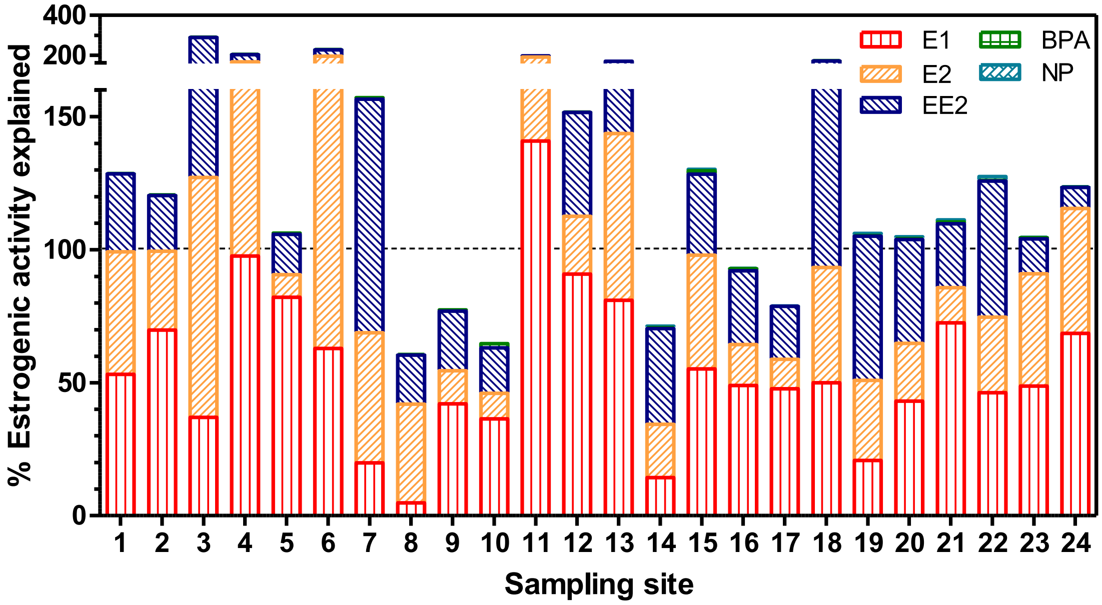

Fig I: Contribution of individual estrogens measured in chemical analysis (EEQ_{chem}, calculated based on relative potencies of the measured estrogens in the bioassay) to the 17β-estradiol equivalent concentrations (EEQ_{bio}, ng/L) measured in the Yeast Estrogen Screen (YES). Values below LOQ were included as LOQ/2.

Mean values from wastewater treatment plant effluent of 24 sites investigated in 2013 and 2014. E1 = estrone, E2 = 17β-estradiol, EE2 = 17α-ethinylestradiol, BPA = bisphenol A, NP = nonylphenol. LOQs were 0.1 ng/L (E1), 0.2 ng/L (E2), 0.3 ng/L (EE2), 1.6 ng/L (BPA) and 1.2 ng/L (NP).

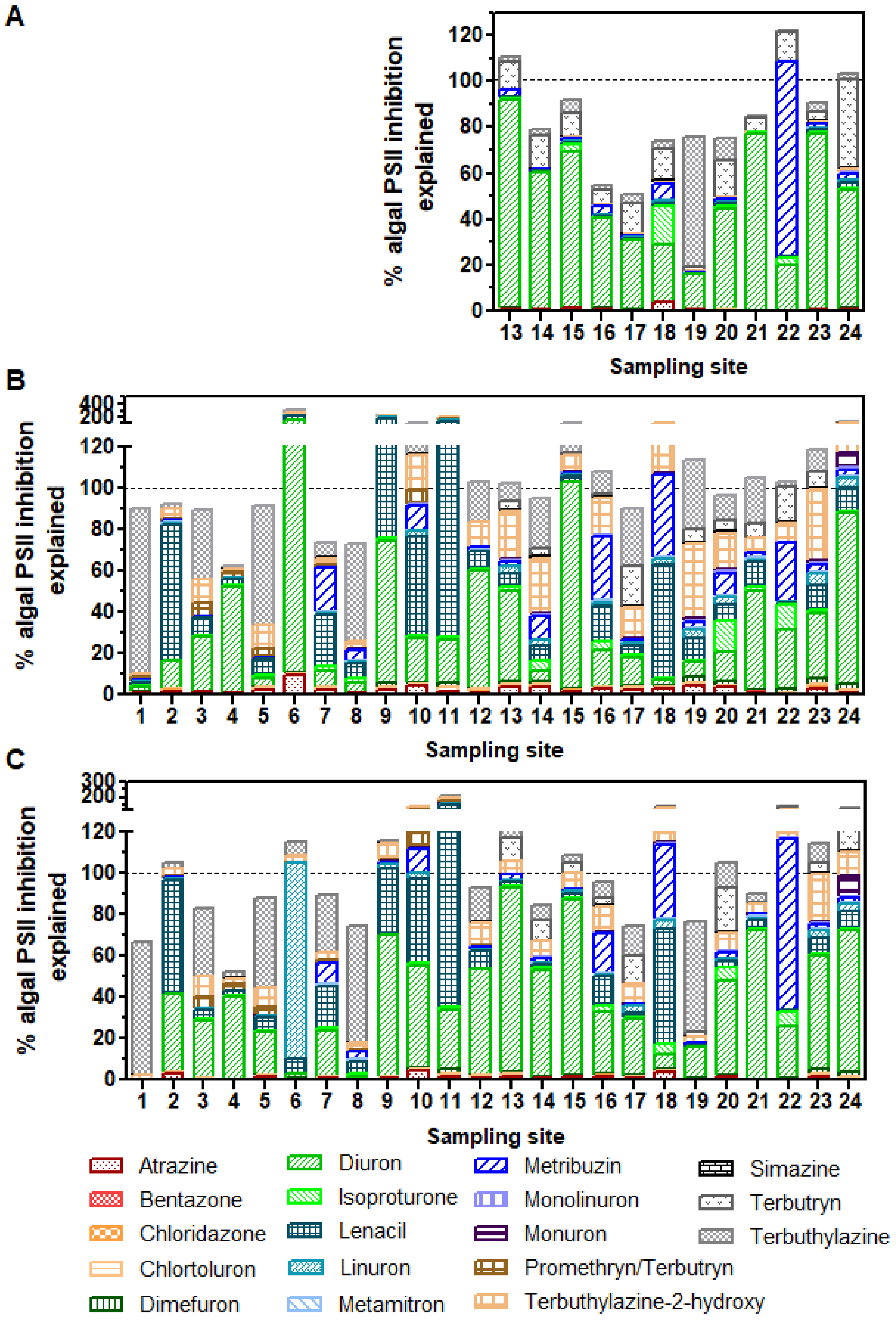

Fig J: Contribution of individual PSII inhibitors measured in chemical analysis (DEQ_{chem}, based on relative potencies of the measured PSII inhibitors in the bioassay) to the diuron equivalent concentrations (DEQ_{bio}, ng/L) measured in the combined algae assay. Values below LOQ were included as LOQ/2.

Mean values from wastewater treatment plant effluent of 12 sites (WWTP effluent, A) and 24 sites (B: upstream, C: downstream). LOQ were determined individually for each sample and are reported in detail in Munz, Burdon [1]. For WWTP effluent, LOQs were not available for all relevant compounds. In this case the LOQ of the next similar sample type was used, i.e. the one from the downstream sampling site.

S8.7 *In vivo* bioassays

Ceriodaphnia dubia reproduction assay

Results of the controls fulfilled the validity criteria: mortality of mothers on the seventh day was ≤ 20% as well as the proportion of males. At least 60% of alive mothers alive have produced a minimum of three broods, and the average number of offspring born per live mother was ≥ 15.

In general, reproduction was enhanced by the tested samples, leading to values above 100%. At one of the four investigated sites (site #6), reproduction was significantly lower downstream compared to upstream (Fig K).

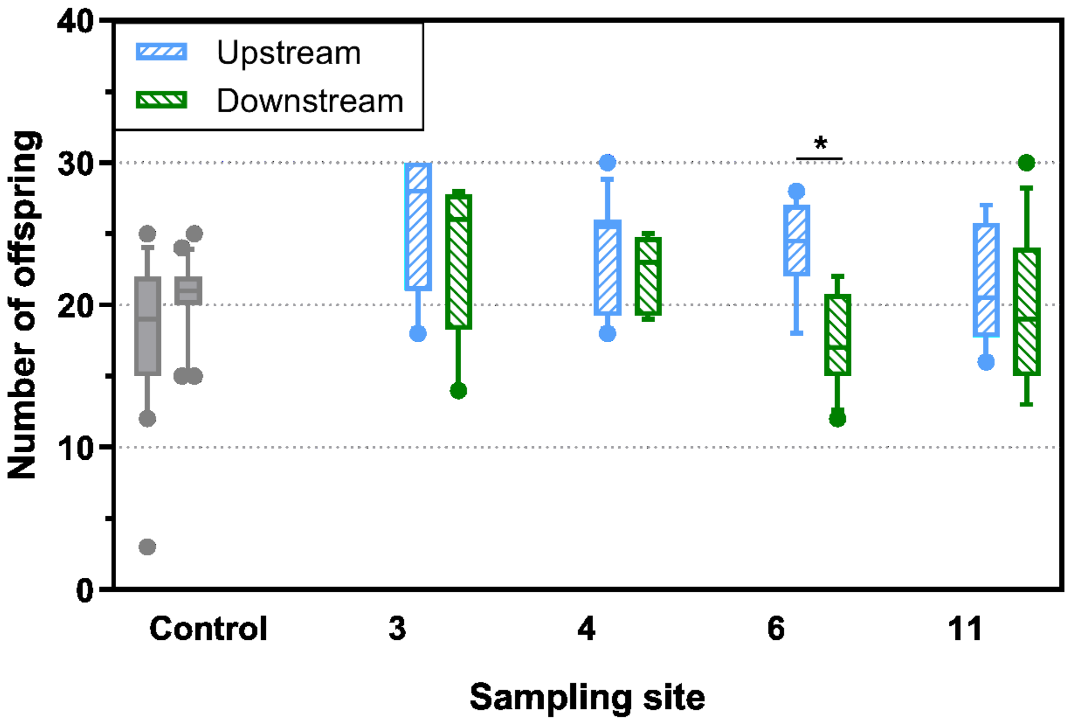

Fig K: Number of offspring of *Ceriodaphnia dubia* after 7 days exposure to river water from 4 sites investigated in 2013.

Box-Whisker plot with the line representing the median, the box the mean 50% of the data and the Whiskers the 10-90 percentile. Dots represent values outside this range. n = 20 (control) and 12 (upstream / downstream), * indicates significant differences (Mann Whitney test). Data are provided in S8_Data.

Gammarus fossarum feeding activity assay

Feeding rate of *G. fossarum* was significantly reduced downstream compared to the upstream site at one of four investigated sites (#1, Buttisholz) (Fig L). At one site, Herisau (site #4), no feeding rate could be determined due to excess sediment in the amphipod cages. Negative feeding rates are partly related to the presence of sediment on the leaves, which could not be fully removed for weight measurements.

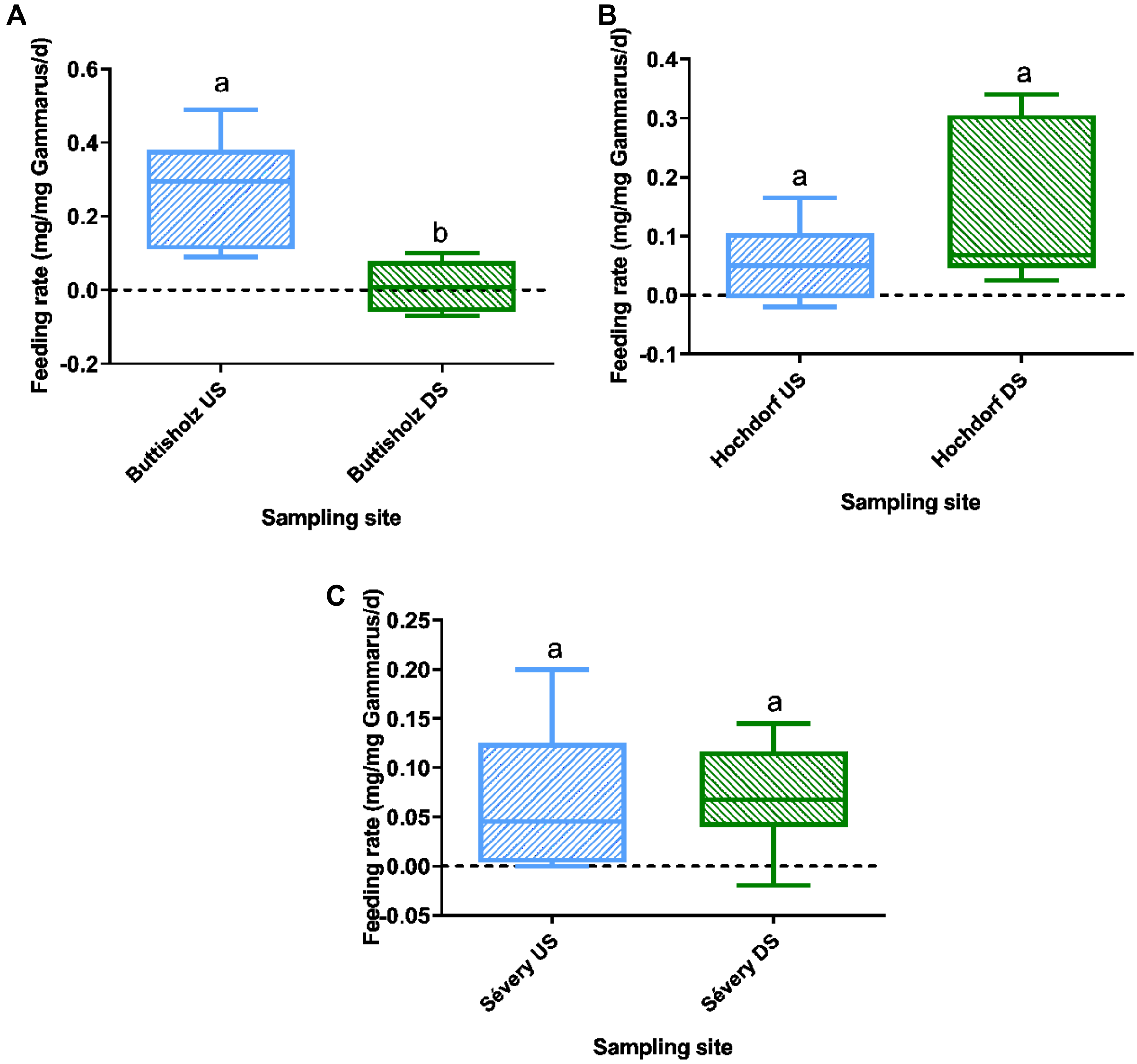

Fig L: Feeding rate of *Gammarus fossarum* (mg / mg Gammarus / d) after 7 days exposure up- (US) and downstream (DS) of three sites in 2013 (Buttisholz, Hochdorf, Sévery).

Box-Whisker plots with the line representing the median, the box the mean 50% of the data and the Whiskers the 10-90 percentile. Dots represent values outside this range. Only feeding rates of live animals were included. n = 4-12. Different letters indicate significant differences (Mann Whitney test). Data are provided in S9_Data.

S8.8 Correlation of effects measured in effluents and river water

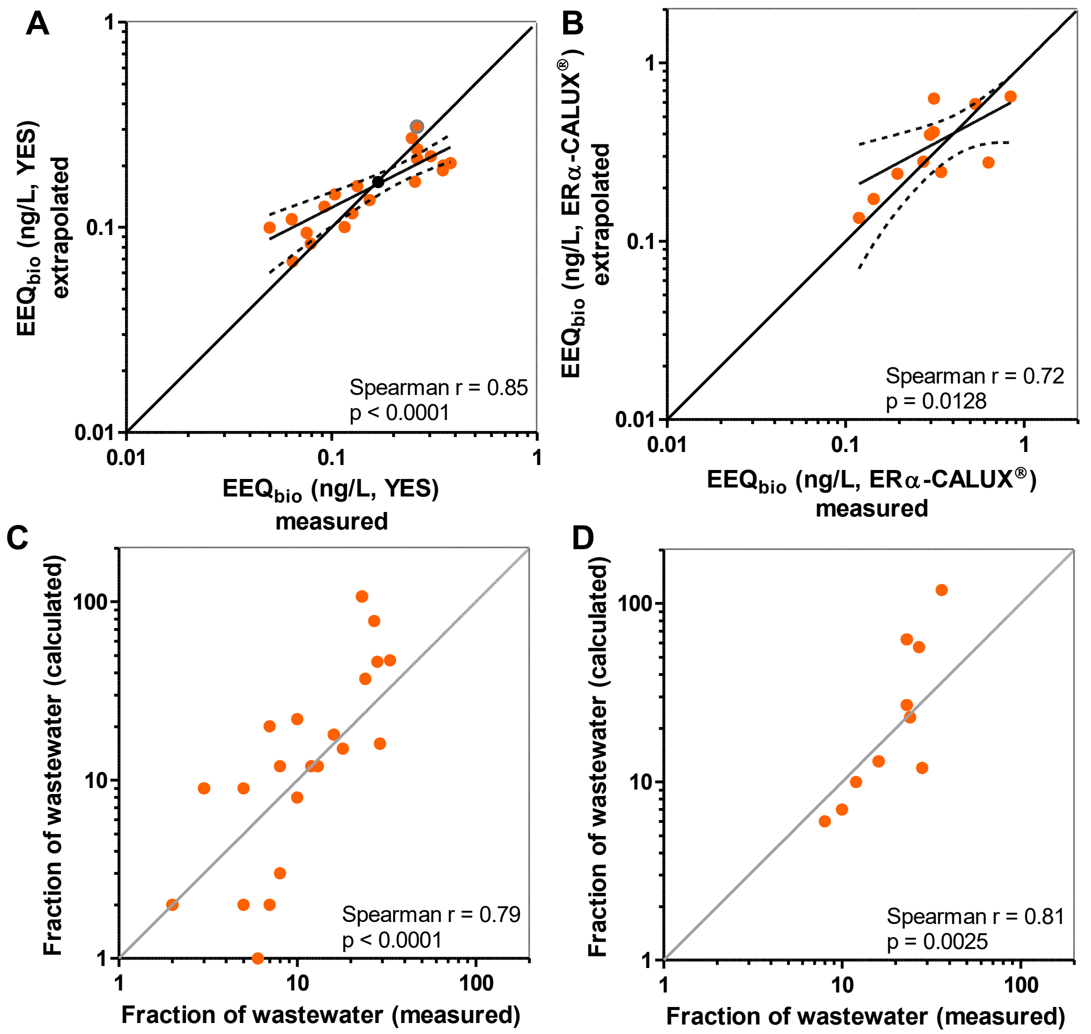

Fig M: Correlation of 17β-estradiol equivalent concentrations (EEQ_{bio}, ng/L) measured downstream in the river in (A) the Yeast Estrogen Screen (YES) and (B) the ERα-CALUX® to the values calculated by measurements in the wastewater multiplied by the respective dilution factor in the river minus the EEQ values measured in the river upstream of the WWTP. C and D display the fraction of wastewater measured using general water chemistry data and calculated based on results of the YES (C) and the ERα-CALUX® (D).

Mean values from 24 sites investigated in 2013 and 2014 (YES) and 12 sites investigated in 2014 (ERα-CALUX®). Black lines indicate non-linear regression ± 95% confidence intervals, grey lines indicate the 1:1 line.

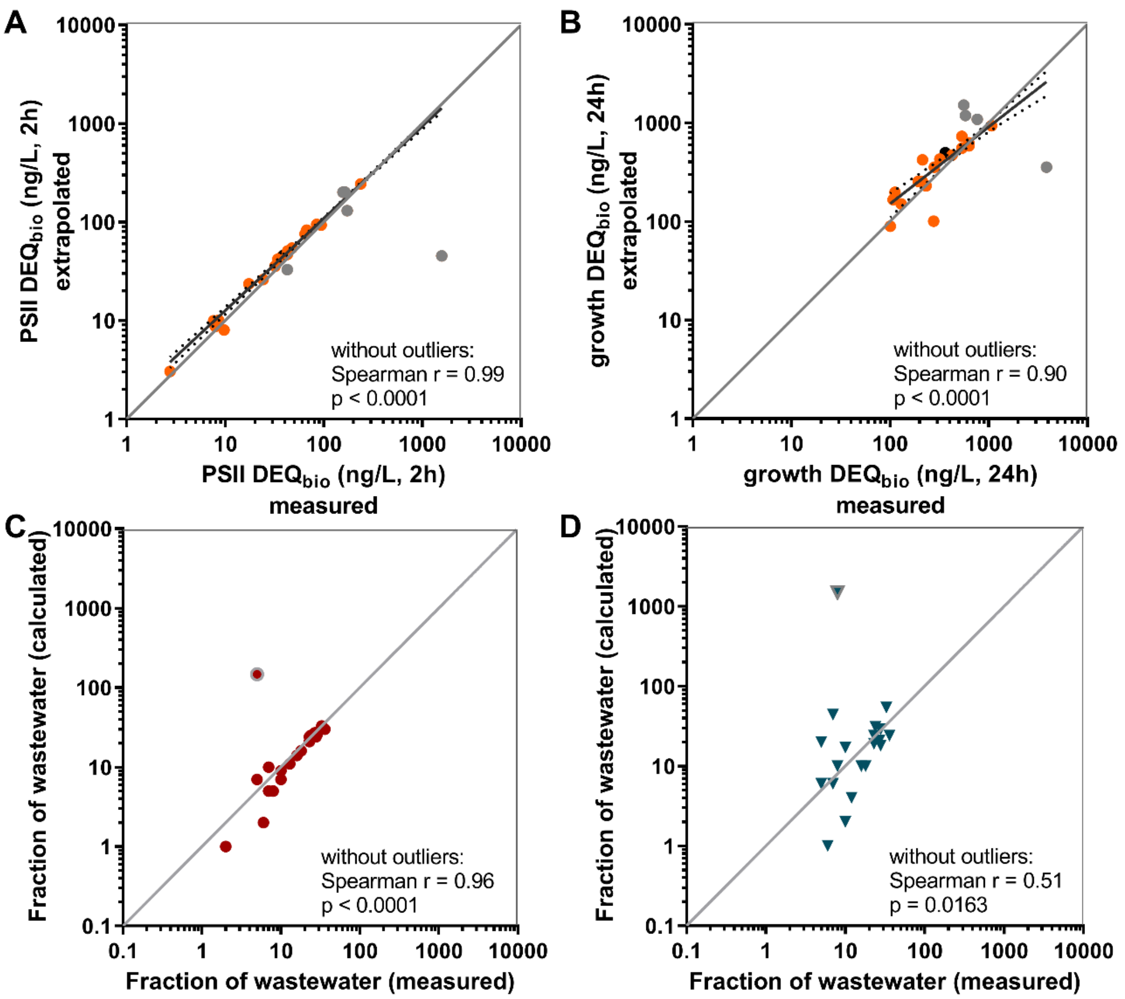

Fig N: Correlation of diuron equivalent concentrations (DEQ_{bio}, ng/L) for (A) photosystem II inhibition and (B) growth inhibition measured in downstream samples in the combined algae assay after 2 h and 24 h respectively to the values calculated by measurements in the wastewater multiplied by the respective dilution factor in the river minus the DEQ_{bio} values measured in the river upstream of the WWTP. C and D display the fraction of wastewater measured using general water chemistry data and calculated based on results for PSII inhibition (C) and growth (D).

Mean values from 24 sites investigated in 2013 and 2014. Outliers are marked in grey (excluded from analysis). Black lines indicate non-linear regression ± 95% confidence intervals, grey lines indicate the 1:1 line.

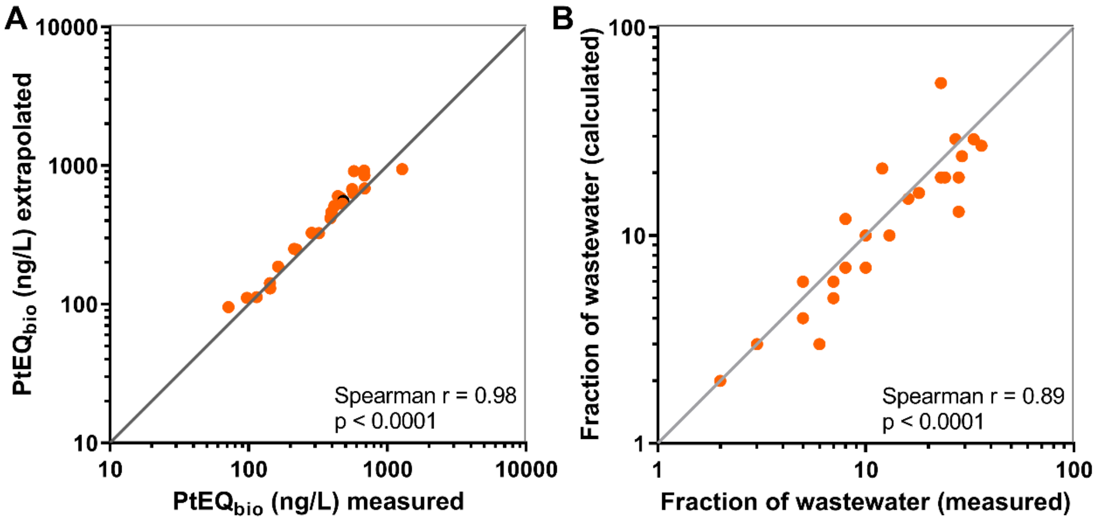

Fig O: (A) Correlation of parathion equivalent concentrations ($PtEQ_{bio}$, ng/L) measured in samples taken downstream in the river to the values calculated by measurements in the wastewater multiplied by the respective dilution factor in the river minus the $PtEQ_{bio}$ values measured in the river upstream of the WWTP. (B) Fraction of wastewater measured using general water chemistry data and calculated based on the bioassay values.

Mean values from 24 sites investigated in 2013 and 2014. Grey lines indicate the 1:1 line.

S8.9 Mixture risk assessment based on bioassay results

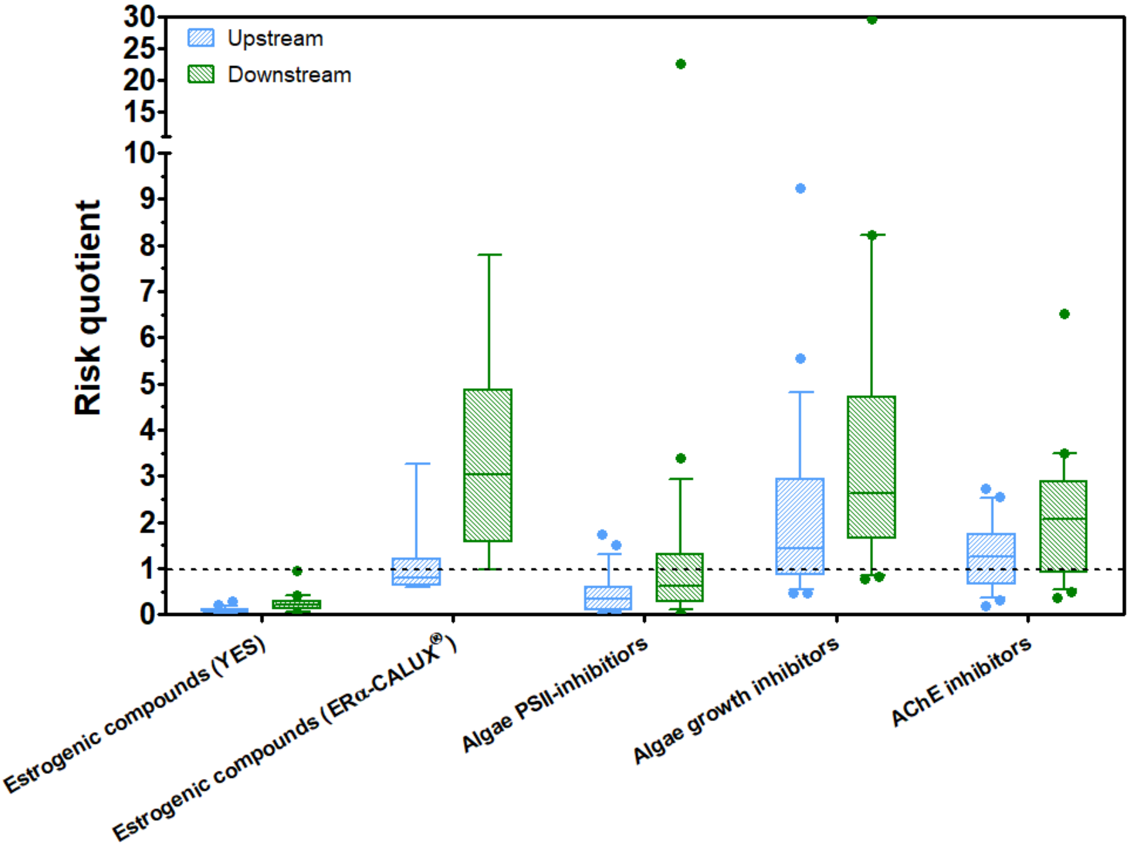

Fig P: Summary risk quotients calculated from bioassay results for four substance groups (estrogenic compounds, algae PSII inhibitors, algae growth inhibitors, acetylcholinesterase inhibitors) measured in the Yeast Estrogen Screen (YES), the ERα-CALUX®, the combined algae assay, and the acetylcholinesterase (AChE) inhibition assay respectively.

Data from 24 sites investigated in 2013/2014 in the river upstream and downstream of the WWTP discharge. Box and Whiskers, 10-90 percentile, dots mark outliers, n = 24 for all bioassays except the ERα-CALUX® (n = 12).

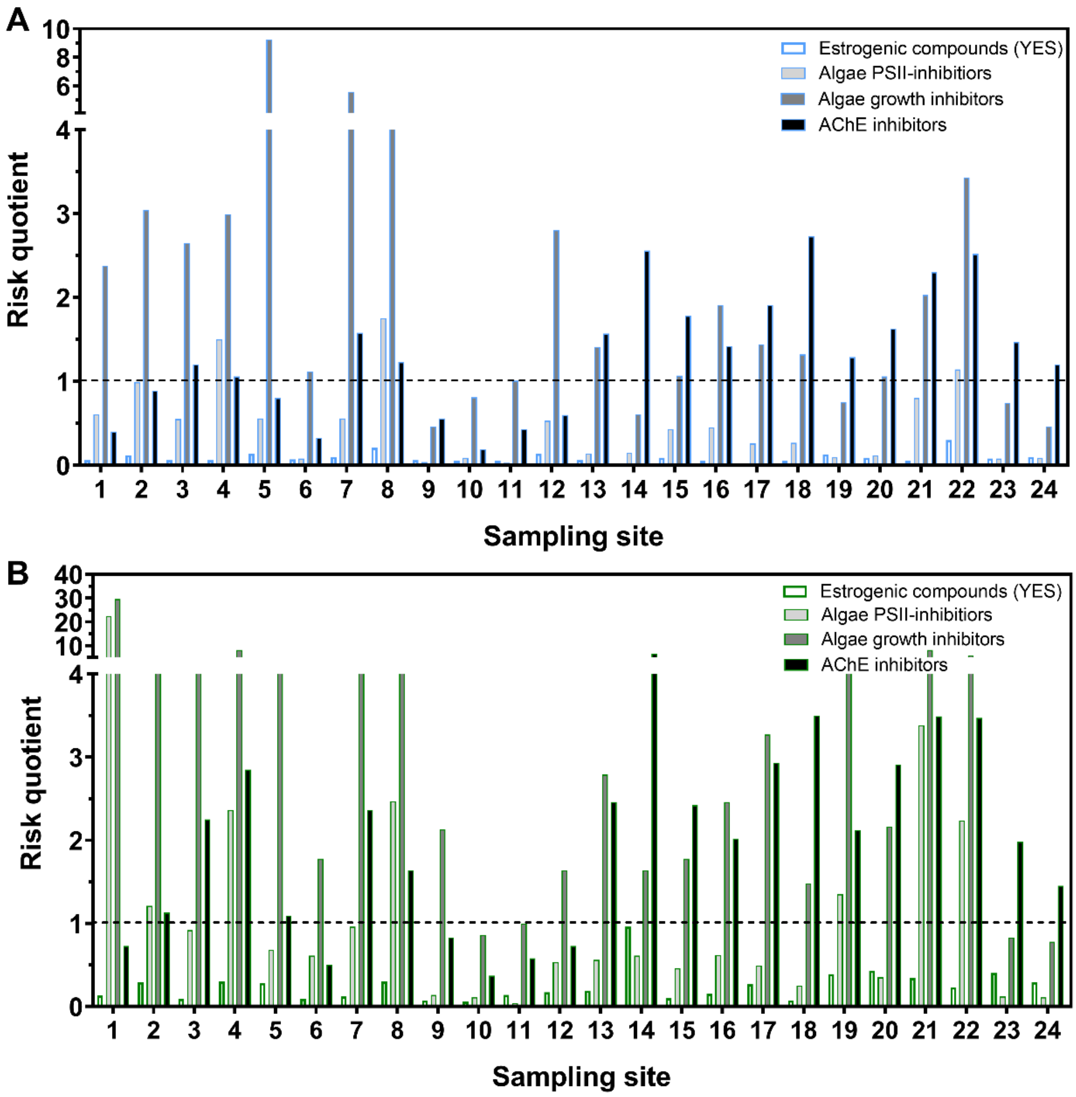

Fig Q: Risk quotients calculated from bioassay results for four substance groups (estrogenic compounds, algae PSII inhibitors, algae growth inhibitors, acetylcholinesterase inhibitors) at 24 sampling sites in A) the river upstream and B) the river downstream of the WWTP discharge.

S8.10 Mixture risk assessment based on chemical analysis results

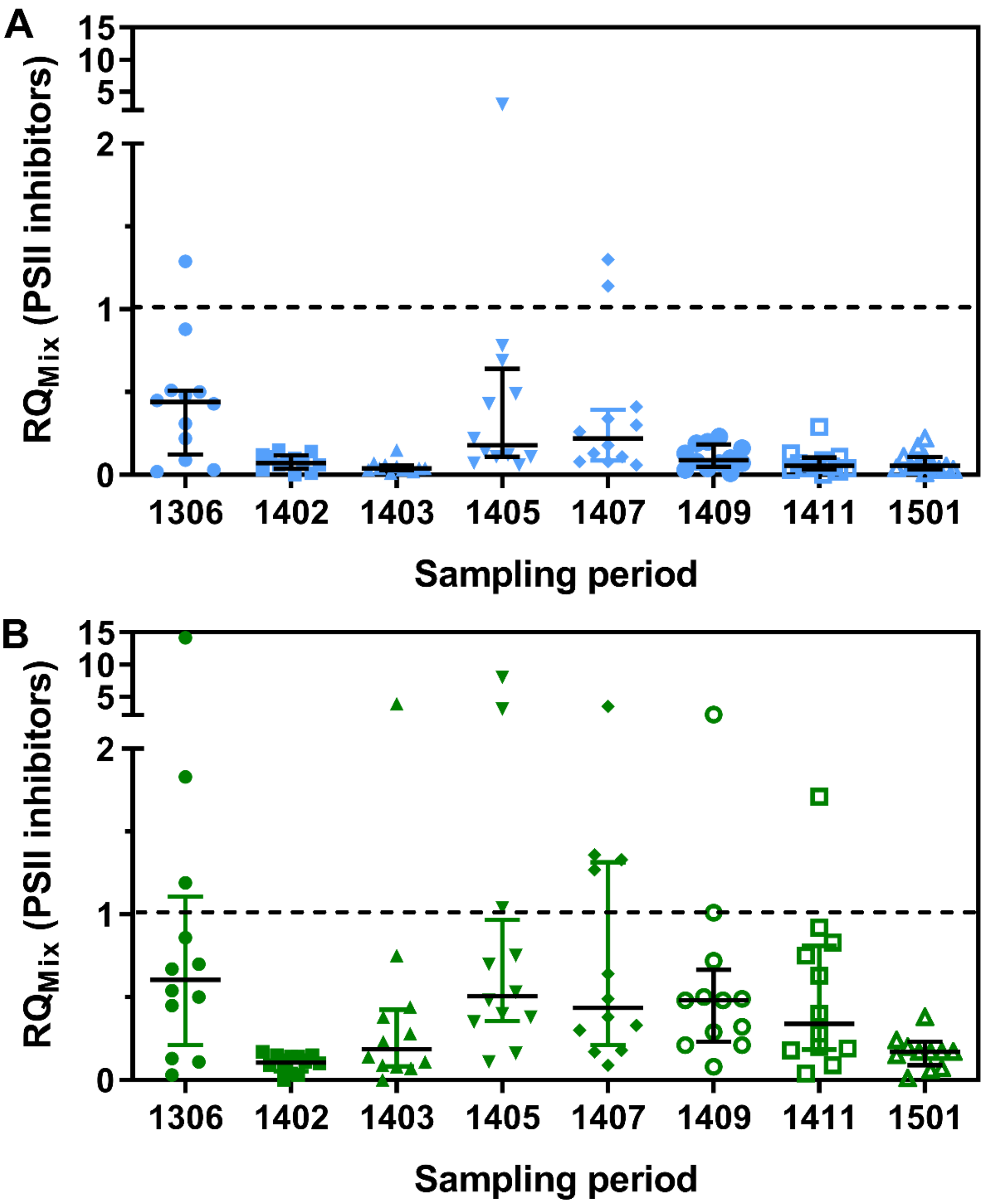

Fig R: Mixture risk quotients (RQ_{mix}) calculated from chemical analysis of photosystem II inhibitors at 24 sampling sites in A) the river upstream and B) the river downstream of the WWTP discharge over 8 sampling events (12 sites per sampling event).

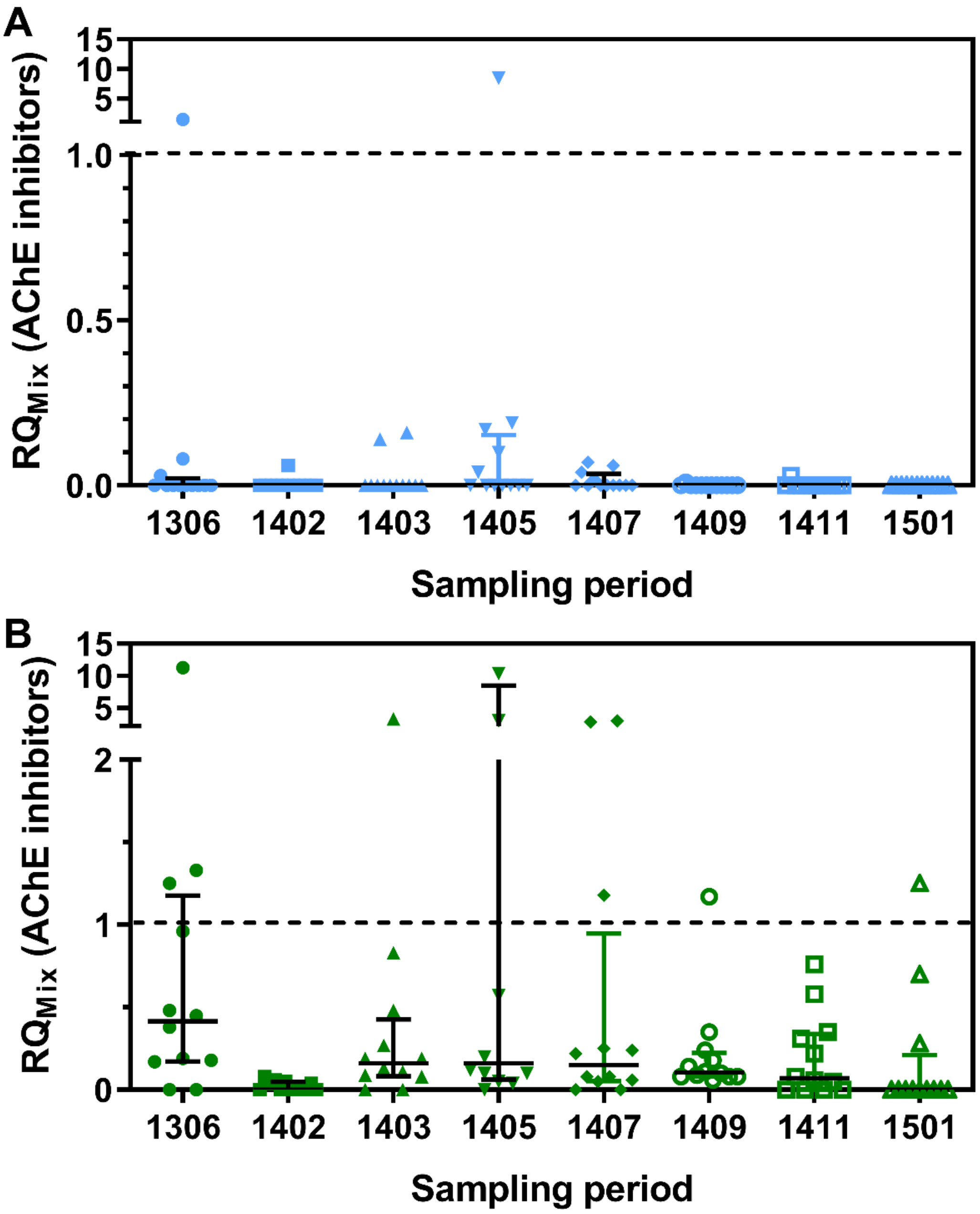

Fig S: Mixture risk quotients (RQ_{mix}) calculated from chemical analysis of acetylcholinesterase inhibitors at 24 sampling sites in A) the river upstream and B) the river downstream of the WWTP discharge over 8 sampling events (12 sites per sampling event).

References

1. Munz NA, Burdon FJ, de Zwart D, Junghans M, Melo L, Reyes M, et al. Pesticides drive risk of micropollutants in wastewater-impacted streams during low flow conditions. Water Research. 2017;110:366-77.
2. Burdon FJ, Reyes M, Alder AC, Joss A, Ort C, Rasanen K, et al. Environmental context and magnitude of disturbance influence trait-mediated community responses to wastewater in streams. Ecology and Evolution. 2016;6(12):3923-39.
3. Bundschuh M, Zubrod JP, Schulz R. The functional and physiological status of *Gammarus fossarum* (Crustacea; Amphipoda) exposed to secondary treated wastewater. Environ Pollut. 2011;159(1):244-9.
4. Franke U. Experimentelle Untersuchungen zur Respiration von Gammarus fossarum in Abhängigkeit von Temperatur, Sauerstoffkonzentration und Wasserbewegung. Arch Hydrobiol (Suppl). 1977;3(4):369-411.
5. Maynard BJ, Wellnitz TA, Zanini N, Wright WG, Dezfuli BS. Parasite-altered behavior in a crustacean intermediate host: Field and laboratory studies. J Parasitol. 1998;84(6):1102-6.
6. Bundschuh M, Pierstorf R, Schreiber WH, Schulz R. Positive effects of wastewater ozonation displayed by *in situ* bioassays in the receiving stream. Environmental Science and Technology. 2011;45(8):3774-80.
7. Maltby L, Clayton SA, Wood RM, McLoughlin N. Evaluation of the *Gammarus pulex in situ* feeding assay as a biomonitor of water quality: Robustness, responsiveness, and relevance. Environmental Toxicology and Chemistry. 2002;21(2):361-8.
8. Water quality -- Determination of chronic toxicity to *Ceriodaphnia dubia.* ISO 20665:2008, ISO 20665 (2008).
9. AFNOR. AFNOR NF T 90-376, Water quality—determination of chronic toxicity to *Ceriodaphnia dubia* in 7 days. Population growth inhibition test. Saint Denis: 2000.
10. Elendt BP, Bias WR. Trace nutrient deficiency in *Daphnia magna* cultured in standard medium for toxicity testing. Effects of the optimization of culture conditions on life history parameters of *D. magna*. Water Research. 1990;24(9):1157-67.
11. Van der Linden SC, Heringa MB, Man HY, Sonneveld E, Puijker LM, Brouwer A, et al. Detection of multiple hormonal activities in wastewater effluents and surface water, using a panel of steroid receptor CALUX bioassays. Environmental Science and Technology. 2008;42(15):5814-20.
12. Rutishauser BV, Pesonen M, Escher BI, Ackermann GE, Aerni HR, Suter MJ, et al. Comparative analysis of estrogenic activity in sewage treatment plant effluents involving three *in vitro* assays and chemical analysis of steroids. Environmental Toxicology and Chemistry. 2004;23(4):857-64.
13. Kunz PY, Simon E, Creusot N, Jayasinghe BS, Kienle C, Maletz S, et al. Effect-based tools for monitoring estrogenic mixtures: Evaluation of five *in vitro* bioassays. Water Research. 2017;110:378-88.
14. Houtman CJ, Van Houten YK, Leonards PEG, Brouwer A, Lamoree MH, Legler J. Biological validation of a sample preparation method for ER-CALUX bioanalysis of estrogenic activity in sediment using mixtures of xeno-estrogens. Environmental Science and Technology. 2006;40(7):2455-61.
15. Spycher S, Mangold S, Doppler T, Junghans M, Wittmer I, Stamm C, et al. Pesticide risks in small streams – how to get as close as possible to the stress imposed on aquatic organisms. Environmental Science and Technology. 2018;52(8):4526-35.
16. Liechti P. Methoden zur Untersuchung und Beurteilung der Fliessgewässer. Chemisch-physikalische Erhebungen, Nährstoffe. Bundesamt für Umwelt (BAFU), Bern, 2010 Umwelt-Vollzug 1005.
17. Escher BI, Aït-Aïssa S, Behnisch PA, Brack W, Brion F, Brouwer A, et al. Effect-based trigger values for *in vitro* and *in vivo* bioassays performed on surface water extracts supporting the environmental quality standards (EQS) of the European Water Framework Directive. Science of the Total Environment. 2018;628-629:748-65.
